# Quantitative microbial taxonomy across particle size, depth, and oxygen concentration

**DOI:** 10.3389/fmicb.2025.1552305

**Published:** 2025-05-23

**Authors:** Paulina Huanca-Valenzuela, Clara A. Fuchsman, Benjamin J. Tully, Jason B. Sylvan, Jacob A. Cram

**Affiliations:** ^1^University of Maryland Center for Environmental Science Horn Point Laboratory, Cambridge, MD, United States; ^2^Center for Dark Energy Biosphere Investigations, University of Southern California, Los Angeles, CA, United States; ^3^Branchpoint Sciences, Los Angeles, CA, United States; ^4^Department of Oceanography, Texas A&M University, College Station, TX, United States

**Keywords:** marine aggregates, East Pacific Rise, oxygen deficient zone, size fractionation, organic matter, microbial communities, microbial diversity

## Abstract

**Introduction:**

Marine particles form in the ocean surface sink through the water column into the deep ocean, sequestering carbon. Microorganisms inhabit and consume carbon in these particles. The East Pacific Rise (EPR) harbors both an Oxygen Deficient Zone (ODZ) and a non-buoyant plume region formed from hydrothermal vents located on the ocean floor, allowing us to explore relationships between microbial community and particle size between a range of environments.

**Methods:**

In this study, we quantified microbial diversity using a fractionation method that separated particles into seven fine scale fractions (0.2–1.2, 1.2–5, 5–20, 20–53, 53–180,180–500, >500 μm), and included a spike-in standard for sequencing the 16S rRNA gene. Size fractionated organic carbon into the same fractions enabled the calculation of bacterial 16S rRNA copies per μg C and per liter.

**Results:**

There was a large increase in the bacterial 16S rRNA copies/ug C and copies/L on particles >180 μm between the upper water column and the deep water column. Though the total concentration of organic C in particles decreased in the deep water column, the density of bacteria on large particles increased at depth. The microbial community varied statistically significantly as a function of particle size and depth. Quantitative abundance estimates found that ostensibly obligate free-living microbes, such as SAR11 and Thaumarcheota, were more abundant in the free-living fraction but also common and abundant in the particulate size fractions. Conversely, ostensibly obligate particle attached bacteria such as members of Bacteroidetes and Planctomycetes, while most abundant on particles, were also present in the free living fraction. Total bacterial abundance, and the abundance of many taxonomic groups, increased in the ODZ region, particularly in the free-living fraction. Contrastingly, in the non-buoyant plume, there were highly abundant bacteria in the 5–20 and 20–53 μm fractions but reduced bacteria present in the 53–180 and 180–500 μm fractions.

**Conclusion:**

Quantitative examination of microbial communities highlights the distribution of microbial taxa unburdened by compositional effects. These data are congruent with existing models which suggest high levels of exchange between particle-attached and free-living communities.

## Introduction

Photosynthesis in the upper ocean is responsible for about 50% of the primary production on earth (Guidi et al., 2009). A fraction of the CO_2_ incorporated into this organic matter sinks to the deep ocean, removing it from the atmosphere for several centuries, and a small fraction of that sinking carbon is entrained in the sea floor removing that carbon from the atmosphere for millions of years (Le Moigne, 2019). The sinking of organic matter into deep waters constitutes a process known as the gravitational biological carbon pump (Le Moigne, 2019). Ocean regions are connected vertically by these marine particles that transport microorganisms from the surface towards the deep (Mestre et al., 2018).

Particulate Organic Carbon (POC) forms through several mechanical and biological processes including zooplankton ingestion/excretion and molting and aggregation of phytoplankton or of smaller particles into marine snow (Simon et al., 2002). Different members of the phytoplankton group contribute differently to the POC export to deep ocean; for example, modeling of carbon export has shown that diatoms could be contributing 40% of carbon export whereas coccolithophores contribute 10% (Jin et al., 2006). Small eukaryotic algae sink in fecal pellets while large eukaryotic algae can sink as single cells (Durkin et al., 2022). Picocyanobacteria aggregates can also contribute to export, but by forming small particles that are aggregated into larger particles below the mixed layer (Cavan et al., 2018; Fuchsman et al., 2019a). The size, and chemical composition of marine particles will depend on the combination of factors that contributed to their formation.

However, not all particles sink; many particles smaller than <53 μm are suspended (Clegg and Whitfield, 1990). Chemical data indicates that suspended and sinking sediment trap material have different isotopic, lipid, pigment and amino acid compositions (Abramson et al., 2010; Altabet, 1988; Wakeham and Canuel, 1988). Older microbial community fingerprinting techniques indicated that communities on sinking particles from sediment traps were significantly different from communities on suspended particles at depth, with trap material more reflective of surface waters (Tamelander, 2013). Recently, amplicon sequencing also indicated differences in microbial communities between suspended and sinking material (Stephens et al., 2024). However, there is connectivity between sinking and suspended particles with disaggregation of sinking particles forming suspended particles (Alldredge, 1998) and aggregation of suspended particles to form sinking particles (Cavan et al., 2018). Additionally, some small particles sink. At the Bermuda Ocean Time series station, in the upper mesopelagic, 39 ± 20% of carbon sinking flux was in 11–63 μm particles (Durkin et al., 2015). This is because density is actually more important to sinking rate than size.

Marine particles can be colonized by microbial communities that use the organic matter for microbial respiration. Metagenomic data from the Hawaii Ocean Time Series indicate that sinking particles are enriched in bacteria that have extracellular peptidases, which degrade proteins, and carbohydrate degrading enzymes as well as large numbers of ABC transporters (Leu et al., 2022). In the absence of oxygen, organic matter can be respired via anaerobic respiration such as denitrification, nitrate reduction or sulfate reduction (Fuchsman et al., 2017; Lam et al., 2009; Raven et al., 2021). The presence or absence of high oxygen concentrations in the water appears to impact the microbial diversity found in particles (Fuchsman et al., 2011; Kellogg and Deming, 2009), and particles themselves have a diffusive boundary layer, and thus can have zones of anoxia despite being in oxic waters (Bianchi et al., 2018; Ploug et al., 1997). Oxygen consumption due to the degradation of organic matter in particles leads to the development of microenvironments within the particles that can support metabolisms that would not be able to take place in the water column, such as denitrification in hypoxic waters (Bianchi et al., 2018; Fuchsman et al., 2019b) and sulfate, and metal reduction in nitrogenous anoxic zones (Raven et al., 2021; Saunders et al., 2019). The formation of anoxic zones inside particles is related to particle size (Ploug et al., 1997).

Most studies tend to assign microbial diversity to two main categories; free-living (FL) or particle-attached (PA). Groups consistently enriched on particles include the phylum Planctomycetes, the order Rhodobacterales (from the Alphaproteobacteria phylum), the phylum Cytophaga-Flavobacteria-Bacteroidetes (CFB) and some types of Gammaproteobacteria such as the order Alteromonadales (Eloe et al., 2011; Fuchsman et al., 2012, 2011; Ganesh et al., 2014; LeCleir et al., 2014; Leu et al., 2022). While some taxa can be classified as strictly FL or PA, other taxa are present in both states (Mestre et al., 2017). In the ocean, marine particles exist in a continuum of sizes through the water column (Nguyen and Harvey, 1997). A simple free-living or particle-attached characterization misses the fact that diffusion into particles is directly related to particle size and surface area to volume ratio, and therefore the interior of large particles may have different redox and nutrient concentrations than found in smaller particles (Ploug et al., 1997). The microbial diversity in individual particles increases with particle size (Stephens et al., 2024). Additionally, particle size directly affects sinking rates and by extension is thereby related to the origin and age of the particle.

A recent approach to fractionating particles, which divided them into 6 size fractions, was used both in the Mediterranean and at 8 open ocean stations during the Malaspina expedition, and showed that different sized particles host different bacterial communities (Mestre et al., 2018, 2017). The diversity was highest in the larger particles (20–200 μm), presumably because larger particles can provide more niches for microorganisms (Mestre et al., 2018, 2017). Mestre et al. (2018) found that communities on 3–20 μm and 20–200 μm size fractions at depth were distinct with the 20–200 μm size fraction more reflecting surface communities. Observations in the lab (Bižić-Ionescu et al., 2018; Datta et al., 2016) and models (Nguyen et al., 2022) suggest that microbial communities likely vary from particle to particle even within a size fraction.

The analysis of microbial amplicon sequences has been limited by the use of relative abundance as an indicator of microbial diversity; relative abundance makes it difficult to directly compare the changes in abundance of microbial groups across different samples. Indeed, relative abundance community data, when analyzed with conventional statistical approaches can produce misleading results, with changes in the actual abundances of one organism creating apparent changes in the opposite direction of other organisms (Gloor et al., 2016). Recently, several studies in microbial ecology have added quantitative elements, such as collecting microscopy data in concert with metagenomic or amplicon based measurements of relative abundance (Fu et al., 2023; Yeh and Fuhrman, 2022), combining quantitative PCR with amplicon sequencing (Shelton et al., 2023; Tettamanti Boshier et al., 2020), and using internal standard spikes (Cram et al., 2024; Jones-Kellett et al., 2024; Tourlousse et al., 2017; Wang et al., 2021). The internal spike approach produces quantitative information by incorporating an artificially designed 16S rRNA gene sequence that amplifies like other 16S rRNA genes, but that is distinct from known 16S rRNA genes of microorganisms (Tourlousse et al., 2017). The use of spike-in standards allows the quantitative estimation of numbers of bacterial 16S rRNA copies per unit of volume and mass (Cram et al., 2024). Additionally, fine scale size fractionation into seven fractions allows us to compare free-living microbes to those inhabiting on small (0.2–1.2, 1.2–5, 5–20 μm), intermediate (20–53, 53–180 μm) and large marine particles (180–500, >500 μm). Compared to the previous work by Mestre et al. (2017, 2018), we have removed the 3 μm size bin and added two larger size fractions (180–500 μm and >500 μm), increasing our chances of capturing larger sinking particles. Thirdly, we size fractionated organic matter into the same size fractions as the fractions analyzed for DNA, so the carbon content of particles in each size fraction were known. The combination of these three techniques has been used in the Chesapeake Bay Estuary (Cram et al., 2024). Here we use this approach for the first time in a depth profile in the open ocean. We specifically address how microbial composition changes among different particle sizes in a water column depth profile above the 9°50’N East Pacific Rise hydrothermal vent field.

### Study site

The 9°50’N, 104°18 W East Pacific Rise (EPR) study site is of particular interest due to the presence of both an Oxygen Deficient Zone in the mesopelagic region and a hydrothermal plume in the bathypelagic region of the same water column. Oxygen Deficient Zones (ODZs) are regions of the ocean that have undetectable oxygen concentration (Revsbech et al., 2009; Tiano et al., 2014). In ODZs, bacteria and archaea use nitrate and nitrite as an electron acceptor instead of oxygen (Lam et al., 2009), leading to N_2_ production by heterotrophic denitrification and the autotrophic anammox process (Babbin et al., 2014; Dalsgaard et al., 2012). ODZs host 30–50% of marine N_2_ production (DeVries et al., 2013). The oxygen content of the Pacific Ocean has been decreasing since the 1980s (Ito et al., 2017), in consequence ODZs are expanding (Evans et al., 2023; Stramma et al., 2008). ODZs have a large anoxic core (O_2_ < 10 nM) surrounded by shells of suboxic (O_2_ < 5 μM), severely hypoxic (O_2_ < 20 μM) and then hypoxic waters (O_2_ < 60 μM). In the geographic core of the ODZ, the vertical width of anoxic waters can be >700 m. However, the vertical width of the ODZ is much smaller at the edges of the ODZ region.

At the ocean floor of 9°50’N EPR is a hydrothermal vent field that underlies the Southern edge of the Eastern Tropical North Pacific (ETNP) ODZ. It is unclear if the Oxygen Deficient Zone in this part of the EPR is truly anoxic or if oxygen is present but not measurable with Seabird sensors typically found on CTD rosettes. In oceanic regions and datasets where determination of oxygen concentration with a STOX sensor (Revsbech et al., 2009) or equivalent is not available, the presence of nitrite at concentrations of 0.5 μM or greater indicates functional anoxia (Banse et al., 2017). Thus, we measured nitrite concentrations here.

Deep sea hydrothermal vents are regions in the ocean floor where ocean water percolates to the subsurface where geothermal processes heat and enrich the water with chemicals and gas, forming a fluid that is released through orifices in the seafloor to the cold deep ocean waters (Dick, 2019). The hydrothermal vent plume at the bottom of the ocean floor at the EPR affects the chemistry and particle composition of the area surrounding the vent and the water column at depth (Dick, 2019). There are two types of plumes: buoyant plumes that rise for hundreds of meters above vents and non-buoyant plumes that spread horizontally away from the vent field. Previous analysis of buoyant and non-buoyant hydrothermal plumes using sediment traps in the EPR, revealed the presence of several microbial groups: Firmicutes, Actinobacteria, Planctomyces, and epsilon proteobacteria, which are now referred to as Campylobacterota (Sylvan et al., 2012). Non-Buoyant plumes evolve as they move away from the hydrothermal vent, with fewer vent related Campylobacterota and more SAR11, S-oxidizing *Thioglobus* Gammaproteobacteria and S-oxidizing *Sulfurimonas* Campylobacterota farther from the vent (Li et al., 2020). In our depth profile, we examined one sample designated as a non-buoyant plume sample that was 163 m from the seafloor above the vent field.

## Methods

### Hydrographic data

Samples were collected as part of the Hot2Cold Vents expedition aboard the R/V *Atlantis* in April 2019, cruise AT42-09. The expedition examined a station in the EPR located at 9°50’N, 104°18.144’W (Fig. S1) which had a bottom depth of ~2,500 m (Fig. S2). A CTD-Rosette was used to collect water samples, and attached sensors recorded oxygen (Seabird 43 Dissolved oxygen sensor), chlorophyll fluorescence (Fluorescence, WET Labs ECO-AFL/FL), beam transmission (Beam Transmission, WET Labs C-Star), temperature, depth, conductivity and salinity. Chlorophyll fluorescence was not calibrated with discrete samples during the cruise. The hydrographic data from the cruise can be found at the R2R rolling deck to repository: https://www.rvdata.us/search/fileset/144040. The EPR station was sampled with CTD casts that targeted 6 different regions: Surface at 4 m, Deep Chlorophyll Maximum (DCM) at 92 m, the mesopelagic (MESO) with two depths (275 m and 316 m), the ODZ with two depths (490 m and 527 m), bathypelagic at 1001 m and Non-buoyant Plume (NBP) at 2369 m (Table 1). The Non-buoyant Plume (NBP) was found using beam transmission data from CTD cast AT420912 (Fig. S2a) and sampled for DNA during that same cast. The temperature of this plume was not elevated compared to the surrounding seawater (Fig. S2b). Due to the volume of water and time needed to size-fractionate samples, only 2–3 depths could be sampled per cast, and samples were obtained over a 6 day period (Table 1). Samples from all the different CTD casts were combined as they were part of the same station.

**Table 1:** Date (local time), coordinates, CTD cast number, Sample category, Depth and Volume of water collected (L) for each water sample.

Date	Latitude	Longitude	CTD Cast	Sample	Depth (m)	Volume water collected (L)
6-Apr-19	9°50.869’N	104°18.144’W	AT4209005	Surface	4	80
				Deep Chlorophyll Maxima (DCM)	92	80
				Mesopelagic (MESO)	275	80
9-Apr-19	9°50.850’N	104°18.157’W	AT4209008	DCM	92	80
				Mesopelagic (MESO)	316	80
11-Apr-19	9°50.874’N	104°18.158’W	AT4209010	ODZ	490	80
				Bathypelagic (BATHY)	1,001	160
11-Apr-19	9°50.874’N	104°18.158’W	AT4209012	ODZ	527	120
				Non-buoyant plume (NBP)	2,369	120

### Sample preparation

Between 80 and 160 L of seawater was collected at each depth by the shipboard rosette (Table 1). Samples were obtained from Niskin bottles by opening the bottom of the Niskin bottle into an acid cleaned bucket. This ensured that particles that had sunk below the spigot of the Niskin bottle (Suter et al., 2017) were included. The water was gravity filtered through a stacked series of 142 mm nylon filters fixed between PVC connectors. The nylon filters were organized and placed one on top of each other in a gradient from the largest pore size at the top towards the smallest size pore at the bottom (500, 180, 53, 20, 5 μm). By pouring the water through the stack of filters, the particles were separated into size fractions on the nylon filters. After filtration, each nylon filter was back rinsed thoroughly using an air powered power water pistol filled with prefiltered (0.2 μm Pall AcroPak 1,500 Capsule with a Supor Polyethersulfone membrane) seawater. The fractionated marine particles were removed from the nylon mesh and collected into a clean bottle (Table 2). All the sampled fractions were divided into two parts: one part was used for DNA analysis and the second part was used to analyze particulate organic C. For DNA analysis, the 5 μm filtrate that passed through all the nylon mesh sizes was filtered onto a Polyethersulfone 1.2 μm filter and a Polyethersulfone 0.2 μm filter using a vacuum manifold. This final 0.2 μm filter collected the free-living microbial fraction. The filters for DNA analysis were placed inside 2 mL sterile screw cap cryogenic tubes, frozen in liquid nitrogen and stored at −80°C until DNA extraction in the laboratory.

**Table 2:** Filter sizes, particles range, filter type and treatment used to recover particles from filters.

Filter size (μm)	Particle range (μm)	Filter type	Treatment used to recover particles from filter
500	> 500	Nylon filter (Gilson Inc.)	Back rinse (nylon filter)
180	180–500	Nylon filter (Gilson Inc.)	Back rinse (nylon filter)
53	53–180	Nylon filter (Gilson Inc.)	Back rinse (nylon filter)
20	20–53	Nylon filter (Gilson Inc.)	Back rinse (nylon filter)
5	5–20	Nylon filter (Spectrum Laboratories)	Back rinse (nylon filter)
1.2	1.2–5	Polyethersulfone filter Sterlitech PES1247100	5 μm filtered that passed through the nylon filters using vacuum manifold
0.2	0.2–1.2	Polyethersulfone filter Sterlitech PES1247100	5 μm filtered that passed through the nylon filters using vacuum manifold

For organic matter analysis, the fractionated resuspended particulate organic matter from each sample was filtered using vacuum via a 1.2 μm nominal pore size, 25 mm diameter, GF/C glass fiber filter (Whatman WHA1822025), that had been pre-combusted at 400°C and pre-weighed using a Sartorius microbalance (precision of 1 μg). Samples for POM analysis were stored in foil packs and also stored at −80°C until analysis in the laboratory.

Filtering may disrupt particles. Due to low organic matter concentrations in the EPR region, to carry out this study, ~120 L of water per depth (Table 1) was filtered in order to collect enough bacteria and particles to be measured. This means that filter clogging was not an issue in this study, but that the large amount of water passing through each filter could theoretically cause disaggregation. However, by passing particles through different filter sizes, and comparing their size distribution using optical scattering before and after filtration, this disaggregation was previously been shown to be minimal (Dougherty et al., 2025).

### Nutrient analysis

Prefiltered (0.2 μm) dissolved nitrate, nitrite, and phosphate from the water samples were analyzed at the Nutrient Analytical Services Laboratory at the Chesapeake Biological Laboratory (CBL) where nitrite plus nitrate were measured by using the Method EPA 353.2. Phosphate by using method EPA 365.1, and Nitrate with the method ASTM D-7781-14. Nitrate concentrations were calculated by subtracting nitrate concentrations from the nitrite plus nitrate numbers. The combined nitrate + nitrite and phosphate concentrations were used to calculate N deficit as described in Chang et al. (2012), a variation on N* that uses N:P ratios from nearby oxic waters, which are ~14, rather than assuming a ratio of 16.

### Particulate organic matter analysis

POM filters were dried at 40°C for >24 h and then were fumed with concentrated HCl for 24 h to remove carbonates. After this treatment the POM filters were packed into silver capsules and sent to the UC Davis Stable Isotope Facility (Davis, CA) to obtain organic C and N concentrations and the natural stable isotopes composition of δ^15^N and δ^13^C. Both carbon and nitrogen were quantified in an Elementar Vario EL Cube coupled with an Isoprim VisION isotope ratio mass spectrometer. A more detailed examination of organic C and N concentrations, ratios and stable isotope data are shown elsewhere (Fuchsman and Cram, 2024).

### DNA extraction and spike-in preparation

DNA was extracted following the Cram et al. (2024) protocol. Frozen samples were extracted from DNA filters using hot SDS lysis followed by phenol-chloroform purification. The DNA concentration of the samples was measured using a Qubit 4.0 (ThermoFisher). Samples were then preserved at −80°C until further analysis.

A synthetic 16S rRNA gene sequence LC140931.1 Synthetic 16S Spike-In (Tourlousse et al., 2017) was synthesized as gBlocks Gene Fragments by Integrated DNA Technologies, Inc. The gBlock was resuspended in a solution of 0.1 mg/mL of PolyA (Roche Diagnostics Deutschland GmbH) in PCR grade water (DEPC-Treated Water Ambion™) and then serially diluted to reach a final concentration of 10^6^ copies/μl following the manufacturer’s instruction.

DNA concentration per sample was diluted using PCR-grade water to reach a concentration of 2 ng/μl DNA (in the case of samples with higher DNA concentrations); then the spike resuspended in PolyA was added to each sample to reach a final concentration of 10^4^ copies/μl or for samples with low DNA concentrations, 5*10^3^ copies/μl.

### DNA sequencing and amplicon bioinformatics

The extracted DNA was sequenced with an Illumina MiSeq by a third-party sequencing company, Molecular Research (MRDNA) Shallowater, Texas, USA. In brief, at MRDNA, 1 ng of sample DNA plus the 10^4^ or 5 * 10^3^ copies of spike were amplified with the HotStarTaq Plus Master Mix Kit (Quiagen, USA) using universal 515FY-926R primers (Parada et al., 2016) with a barcode added to the forward primer. Amplification conditions were 94°C for 3 min followed by 30 cycles of 94°C for 30 s, 53°C for 40 s and 72°C for 60 s. There was a final elongation step of 72°C for 5 min. Following amplification, PCR products were checked for length and intensity on a 2% agarose gel. Samples were then pooled together in equal proportions and purified using Ampure XP beads. These pooled cleaned products were sequenced on a MiSeq instrument (2×250 base pair reads) following manufacturers guidelines. Samples were returned to our group as FASTQ files.

The EPR sequenced amplicons were processed following the same pipeline described in Cram et al. (2024), which will be briefly summarized here. The sequenced amplicons were processed using the DADA2 pipeline (Callahan et al., 2016). The primers were removed from the sequences using Cutadapt (Martin, 2011), with the following modification, the filterandtrim() algorithm was used to retain sequences with < three errors in the forward read and fewer than five errors on the reverse read. Then DADA2 was used to dereplicate, obtain error rates, and merge forward and reverse reads. The taxonomy of each ASV was assigned using the Silva database version 132 released Dec 2017 (Quast et al., 2013). This database was modified by adding the spike-in sequences (Cram et al., 2024). Data were analyzed using the R software for statistical analysis (V4.0.2) using the packages Vegan, Phyloseq for data analysis, Dplyr for data curation and ggplot2 to visualize data.

### Calculating taxon abundance

The taxon abundance (16S + 18S rRNA gene copies/L) of each microbial group in each sample was estimated by normalizing with the spike in read numbers, DNA extracted and volumes filtered, following Equation 1. In two cases, no spikes were observed in our sample. Our lowest sequence depth was 7,052 sequences in the 0.2 μm 1,000 m sample, which corresponded to no observed spikes, presumably because 10^4^ copies was not enough spike to detect in every sample. In the other sample without spiked standards at 5 μm 490 m, there were 25,162 read copies, which is large enough that we would have expected to see spiked standards in this sample. We fear that spiked standards were not added to this sample. For these two cases, we used a spike count of one, rather than zero, in order to show the taxonomic information for these samples.


(1) $$\begin{array}{l} \text{Normalized Taxon Abundance}\;(\text{Copies}/L)=\frac{\mathrm{ASV}\;\text{Reads}}{\text{Spikes Reads}}\\ {}*\frac{10^{5}\text{Spike Reads}}{l\;\mathrm{ng}\;\mathrm{DNA}}*\frac{\mathrm{DNA}\;\text{Extracted}(\mathrm{ng})}{\text{Volume Filtered}(L)}\\ {}*\frac{\text{Total Volume of Rinse water}(L)}{\text{Volume of Rinse water used for}\;\mathrm{DNA}\;\text{filter}(L)} \end{array}$$

The quantitative examination of microbial amplicons in particles using spike-in standards allowed for calculating the number of bacterial and archaeal gene copy numbers. Using these same primers, picocyanobacteria, which are known to have only one copy of the 16S rRNA gene, closely corresponded to cell counts from flow cytometry data (Jones-Kellett et al., 2024). When examining whole microbial communities, microbial abundance is directly related to 16S rRNA copy numbers, but the copy numbers are often somewhat higher than microscopic count data (Cram et al., 2024). Therefore, we have been careful herein to report 16S rRNA gene copy abundances, rather than cellular abundances.

### Calculating particle carbon content

To estimate the amount of organic carbon present in the original volume of water sampled, total observed μg C was normalized to the volume of the water that went through the stacked set of nylon filters and the fraction of water that was rinsed through the GF/F filter, using Equation 2.


(2) $$\begin{array}{l} \text{Organic}\;C\;\text{Concentration}(\mathrm{\mu g}/L)={\frac{\text{Carbon}\;\;\text{Content}}{\text{Volume}\;\;\text{Filtered}(L)}}^{*}\\ \;\frac{\text{Total}\;\;\text{Volume}\;\;\text{of}\;\;\text{Rinse}\;\;\text{Water}(L)}{\text{Volume}\;\;\text{of}\;\;\text{Rinse}\;\;\text{water}\;\;\text{used}\;\;\text{for}\;\;\mathrm{POM}\;\;\text{Measurement}(L)} \end{array}$$

Microbial abundance was normalized to carbon weight by dividing the taxon abundance by the carbon composition of marine particles following Equation 3.


(3) $$\begin{array}{l} \text{Normalized}\;\;\text{Taxon}\\ \;\;\text{Abundance}(\text{copies}/\mathrm{\mu g})=\frac{\text{Taxon}\;\;\text{Abundance}(\text{copies}/L)}{\mathrm{C\mu g}/L} \end{array}$$

We conducted a redundancy analysis to see if there was a statistical effect of particle size (μm) or depth (m) on community structure. In order to analyze the relative similarities in community structure between all samples, microbial abundance was log-transformed and depth was treated as continuous data. Samples were color and shape coded based on size class and the depth from which they were collected. In particular, we used an RDA of form ‘log(OTU_Abundances) ~ log(Depth) + log(Depth)^2 + log(Size) + log(Size)^2 + log(Depth) * log(Size).’ This model looks for non linear effects of depth and size on community structure as well as interactions between depth and size effects. We tested for statistical significance using an ANOVA-like Permutation Test for Redundancy Analysis.

## Results

### Hydrographic and biochemical context for the EPR

**Figure 1:**
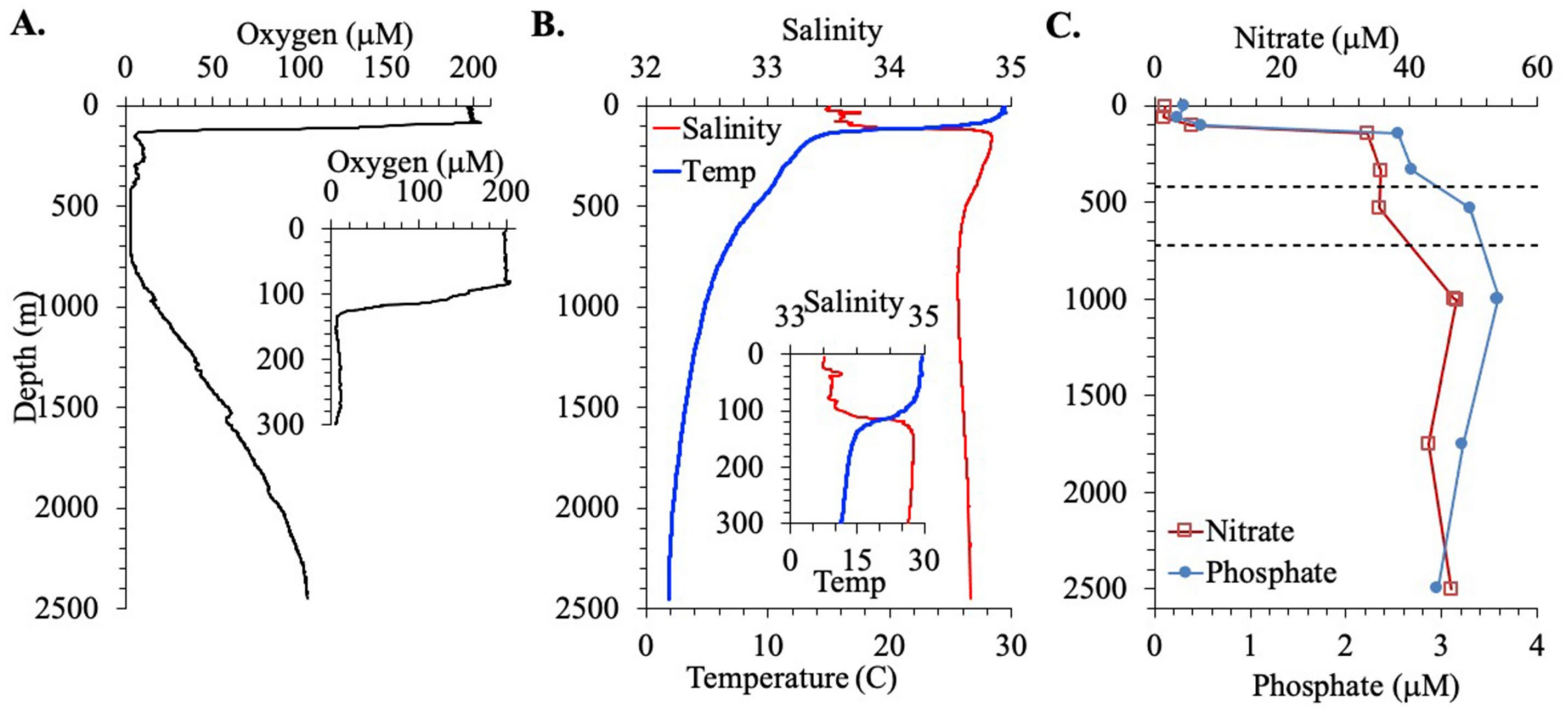
Hydrographic parameters for the EPR station. **(A)** Oxygen concentrations for the full water column with an inset for the top 300 m. **(B)** Salinity and temperature for the full water column with an inset for the top 300 m. **(C)** Nitrate and phosphate concentrations. Dashed lines indicate the boundaries of the ODZ.

**Figure 2:**
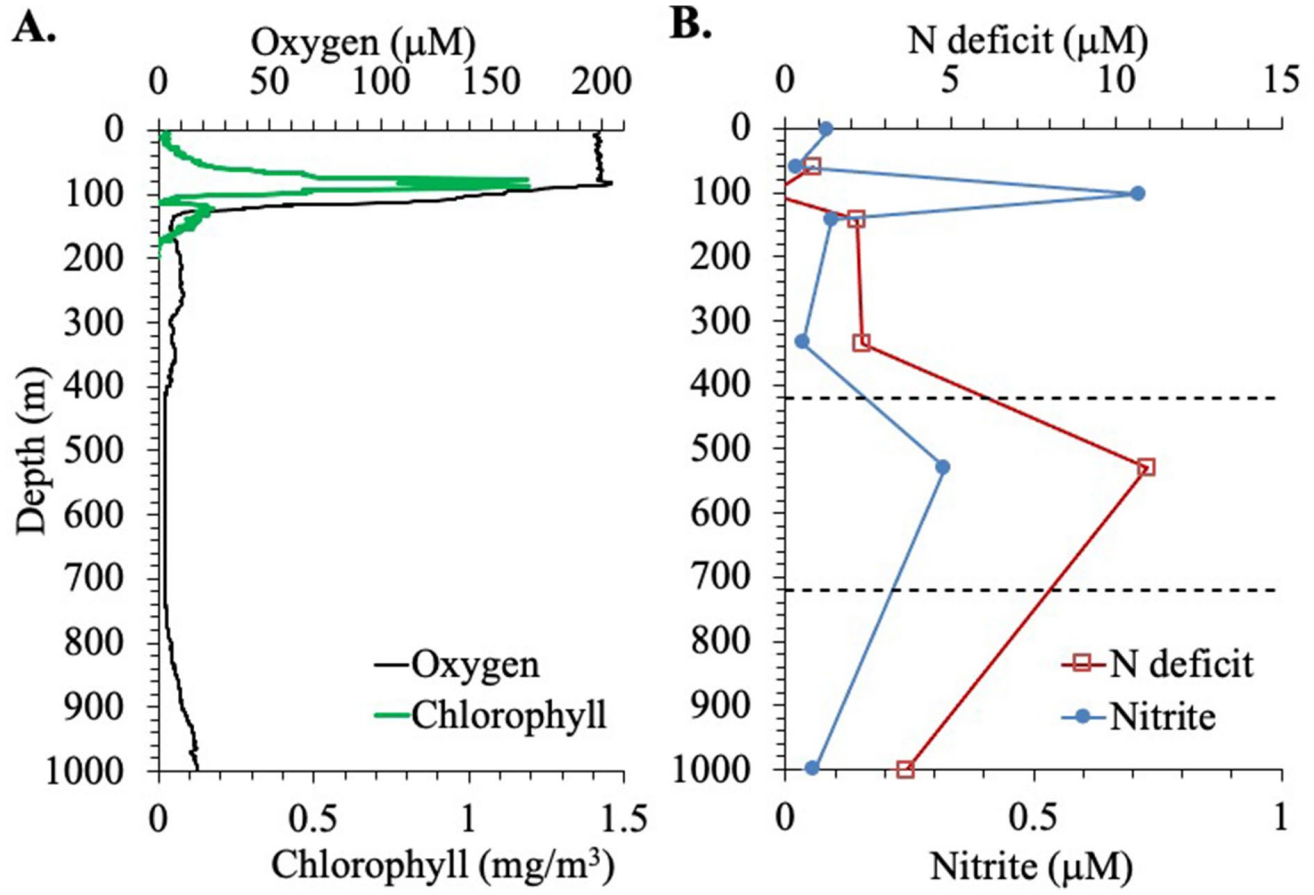
Biochemical profiles at the EPR station. **(A)** Oxygen (μM) and Chlorophyll concentrations (mg/m^3^). **(B)** Nitrite concentration and nitrogen deficit, through the top 1,000 m of the water column. Dashed lines indicate the boundaries of the ODZ.

First we examine the hydrographic and biogeochemical context for this station. Salinity (33.5), temperature (29.5°C) and oxygen concentrations (198 μM) were constant for the top 75 m of the water column. Then values changed sharply in the 75–140 m region with salinity increasing to 34.8 and temperature and oxygen concentrations decreasing to 14.5°C and 6 μM, respectively, (Figure 1). The concentration of chlorophyll a was low in the top 10 m (~0.1 mg/m^3^), and there was a deep primary chlorophyll maximum at 60–80 m of 1.3 mg/m^3^ (Figure 2A). Between 140 and 400 m depth, oxygen concentrations fluctuated between 5 and 10 μM and temperature slowly decreased to 10°C. In the area between 400 and 700 m, the oxygen concentration was constant at 2.6 μM (Figure 1). This value may have been the sensor detection limit. While other Seabird sensors can reach 1 μM, detection limits can depend on the individual instrument (Kwiecinski and Babbin, 2021). Thus, it is not possible to tell if the oxygen depleted region was anoxic. Below this region, oxygen concentrations increased again, reaching 17 μM at 1000 m while temperatures continued to decrease, reaching 4.7°C. In the deepest waters, oxygen concentrations reached 100 μM and temperatures decreased to 1.9°C (Figure 1).

The presence of nitrite was observed in the water column in the EPR (Figure 2B). Nitrite accumulation was observed at two peaks: a primary nitrite maximum at 103 m, where nitrite reached 0.71 μM, and a secondary nitrite maximum at 0.32 μM at 530 m (Figure 2B). Banse et al. (2017) used 0.5 μM nitrite as a cut off for using nitrite to indicate true anoxia. The secondary nitrite maximum found here is close but less than this cut off, but higher values could have missed due to the sampling scheme. Nitrate and phosphate were also measured in the EPR water column. Nitrate was ~1.5 μM in the top 60 m and then increased to 5 μM at 100 m and to 40 μM at 1000 m depth (Figure 1C). Phosphate generally had a depth profile pattern similar to nitrate with 0.3 μM phosphate in the surface, 0.5 μM at 100 m and a maximum of 3.6 μM at 1000 m (Figure 1C). In addition, a nitrogen deficit of 10.9 μM at 530 m depth in the ODZ region was calculated from this nutrient data (Figure 2B). Outside the ODZ, the nitrogen deficit was <2 μM. These N deficit values are typical for those measured in the ETNP ODZ (Fuchsman et al., 2018), and could reflect local denitrification under anoxic conditions, however the nitrogen could also have been removed elsewhere and this low N water advected into the local system (Peters et al., 2018). Thus, the suboxic versus anoxic condition of the water in the EPR ODZ cannot be determined by this chemical data alone.

### Concentrations of carbon and microbial 16S rRNA in particles

**Figure 3:**
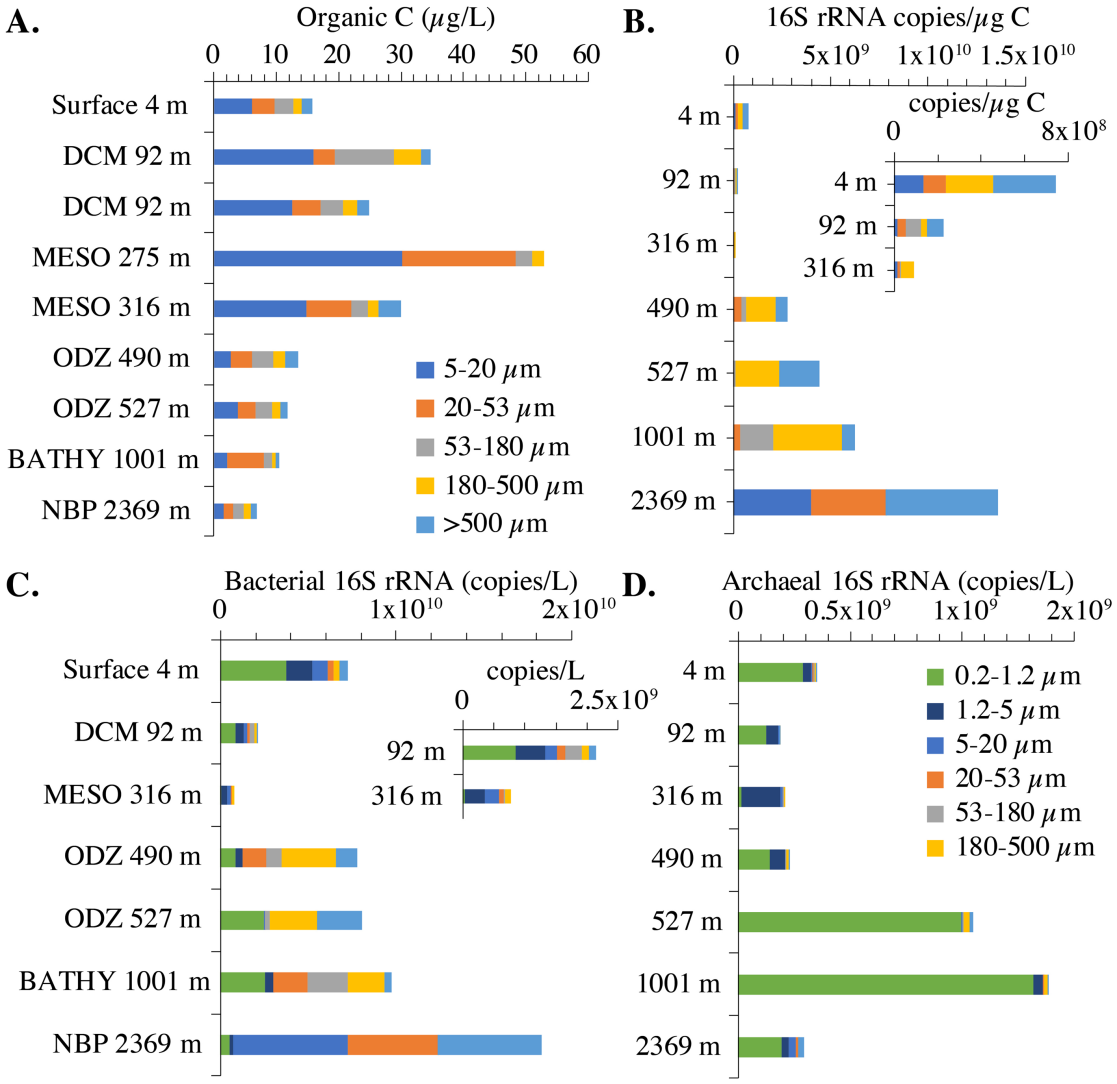
Organic C concentrations and bacterial 16S rRNA copies across size fractions in the EPR. **(A)** Particulate organic carbon concentrations in marine particles. The >500 μm organic matter fraction for 275 m is missing. **(B)** Bacterial 16S rRNA copies/μg organic C across seven size fractions. Inset: Bacterial 16S rRNA copies/μg organic C zoomed in on 4, 92 and 316 m. **(C)** Bacterial 16S rRNA copies/L across seven size fractions. Inset: Bacterial 16S rRNA copies/L zoomed in on 92 and 316 m. **(D)** Archaeal 16S rRNA copies/L across seven size fractions. For 16S rRNA copies, the 275 m and one 92 m sample were not included because too many size fractions were missing. Additionally, 1.2–5 μm size fraction is missing from the 527 m sample, 53–180 μm fraction is missing from the 4 m sample, and the >500 μm fraction is missing from the 316 m sample. The 5–20 μm size fraction for 490 m was not included. DCM, deep chlorophyll maxima, ODZ, oxygen deficient zone, MESO, mesopelagic, BATHY, bathypelagic; NBP, non-buoyant plume.

In the surface sample (4 m), the total combined POC concentration was 16 μg/L. Two samples were obtained in the DCM (92 m) three days apart (Table 1), and their total combined POC concentrations varied between 25 and 35 μg/L. The highest total combined POC concentration (54 μg/L org C) was at 275 m (Figure 3A), and it was still elevated at 316 m (30 μg/L). In the ODZ and at 1000 m, the total combined POC concentrations were 14 μg/L or less (Figure 3A). The non-buoyant plume (2,369 m) contained the smallest amount of total organic C (8 μg/L). At depths above the ODZ, particulate organic C (POC) concentrations were highest in the 5–20 μm fractions but there were still significant concentrations in the 20–53 μm and the 53–180 μm fractions (Figure 3A). However, in the ODZ (490 and 527 m) and below, the POC concentrations in the 5–20 μm fraction were particularly reduced (Figure 3A). In fact, at 1000 m, POC concentrations were highest in the 20–53 μm fraction (Figure 3A). Thus in the top 350 m of the water column, the POC concentrations in small particles (<20 μm) were larger than the concentrations in intermediate particles (20–180 μm), which in turn were larger than the concentration in large particles (>180 μm). However, at depths >350 m, intermediate sized particles often had the largest concentrations of organic C (Figure 3A).

**Figure 4:**
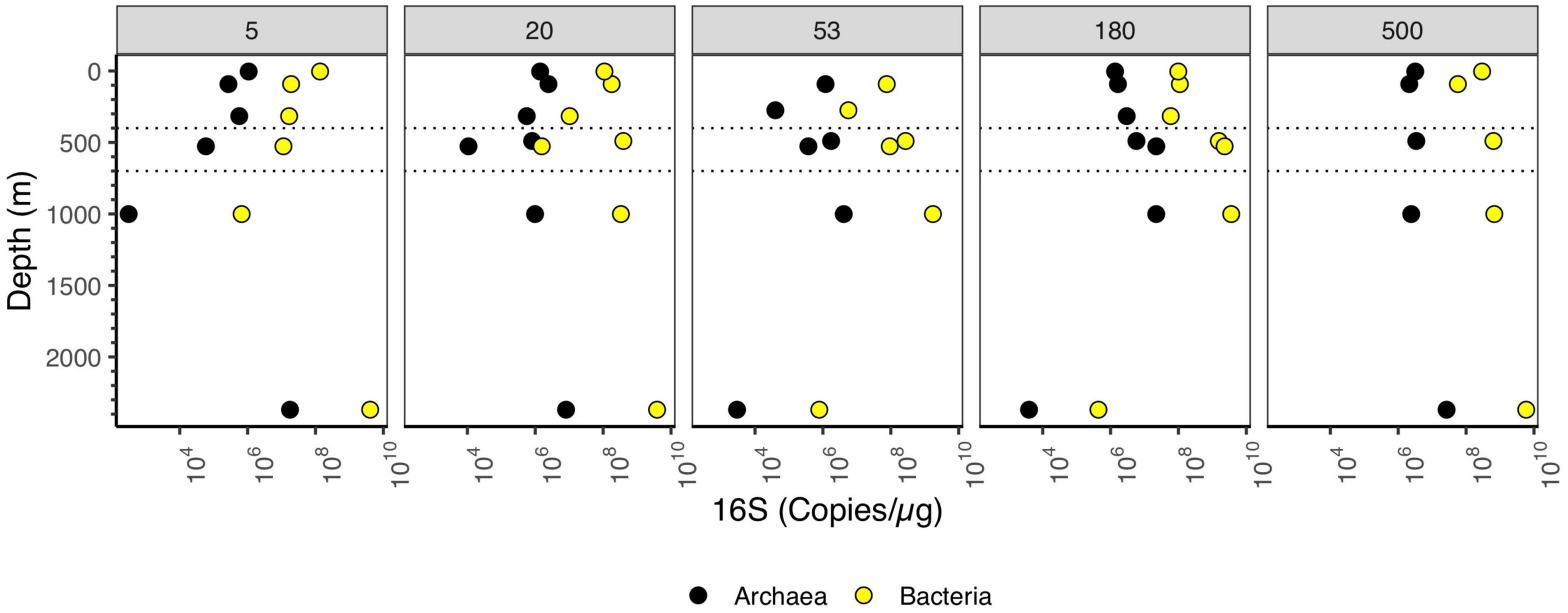
Depth profile of 16S rRNA gene copy numbers per μg organic C, aggregated to domain level. Each panel represents particle size (in μm). The Y axis corresponds to depth (m), and the X axis corresponds to 16S rRNA gene copies/μg organic C. Dashed lines indicate the boundaries of the ODZ.

We examined the abundance of archaea and bacteria on particles in two ways: in 16S rRNA copies per L, and copies/μg org C. When the size fractions were examined together, one can see different patterns in the upper and lower water column. In the upper water column, the numbers of bacterial 16S rRNA copies/μg org C on >180–500 μm and >500 μm particles was much smaller (10^8^ copies/μg org C) than found in the deeper water column (10^9^ copies/μg org C) (Figure 3B). However, for the smaller 5–20 μm particle size, the abundance of 16S rRNA gene copies/μg org C decreased as depth increased; bacterial copies/μg org C abundance was higher in surface (10^8^ copies/μg org C), decreasing to 10^7^ copies/μg org C in the mesopelagic and ODZ and decreasing again to 10^5^ copies/μg org C at 1000 m (Figure 4). However, values then increased to ~10^9^ copies/μg org C in the Non-buoyant plume at 2,369 m depth (Figure 4). The Non-buoyant plume had a fundamentally different structure of bacteria on particles, with extremely high numbers of 16S rRNA copies/μg org C in the 5–20, 20–53, and >500 μm fractions (>10^9^ copies/μg org C), but extremely low numbers in the 53–180 and 180–500 μm fractions (10^5^ copies/μg org C) (Figures 3, 4). For these copies/μg org C numbers, it is difficult to separate changes in 16S rRNA copies from changes in organic C concentrations. Therefore, we also examine 16S rRNA copies/L for each size fraction, which indicates whether the numbers of microbes are increasing or decreasing irrespective of changes in organic C.

**Figure 5:**
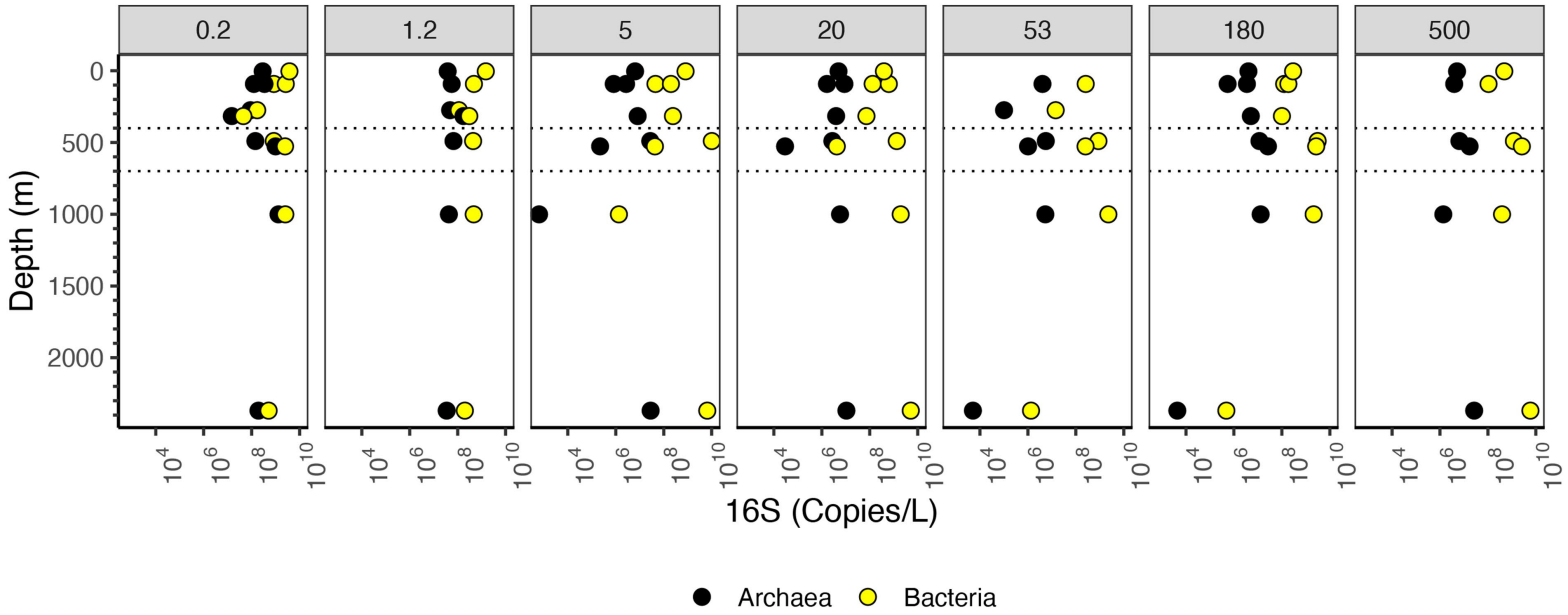
Depth profile of 16S rRNA gene copy numbers per L, aggregated to domain level. The Y axis corresponds to depth (m), and the X axis corresponds to 16S rRNA gene copies/L. Dashed lines indicate the boundaries of the ODZ.

In the upper water column, bacterial 16S rRNA copies per L were higher in the free-living fraction and smaller particles and lower in large particles. However, in the ODZ and below, bacterial 16S rRNA copies per L were higher in intermediate and large particles (Figure 3C). The mesopelagic samples (316 m) had particularly low numbers of bacterial 16S rRNA copies overall, which was surprising since the organic C concentrations were particularly high at this depth (Figure 3A). Archaeal 16S rRNA copies per L, on the other hand, were primarily found in the free-living and 1.2–5 μm fractions (Figure 3D). Archaea abundance was always lower than bacteria abundance usually by one or two orders of magnitude, but the depth profiles for bacteria and archaea had similar shapes (Figure 5).

The number of bacterial 16S rRNA copies per L in the different size fractions varied with depth. In the free-living size fraction, there were high 16S rRNA copies per L in the surface (~10^9^ bacterial 16S rRNA copies/L), which decreased in the mesopelagic oxycline (10^7^ copies/L), but increased again in the ODZ (~10^9^ bacterial 16S rRNA copies/L) (Figure 5). Similar to the copies/μg org C data, the numbers of bacterial 16S rRNA copies/L in the 5–20 μm fraction decreased from the surface to the oxycline and decreased further in the bathypelagic. Also similar to the copies/μg org C data, in the non-buoyant plume, the highest copy numbers of 16S rRNA were found in the 5–20 and 20–53 μm fractions and the >500 μm fractions (~10^9^ copies/L); however, 16S rRNA copy numbers were quite low (~10^6^ copies/L) in the 53–180 and 180–500 μm fractions. Thus, the general trends were similar between copies per L and copies/μg org C data.

### Abundance of bacterial taxa on particles

**Figure 6:**
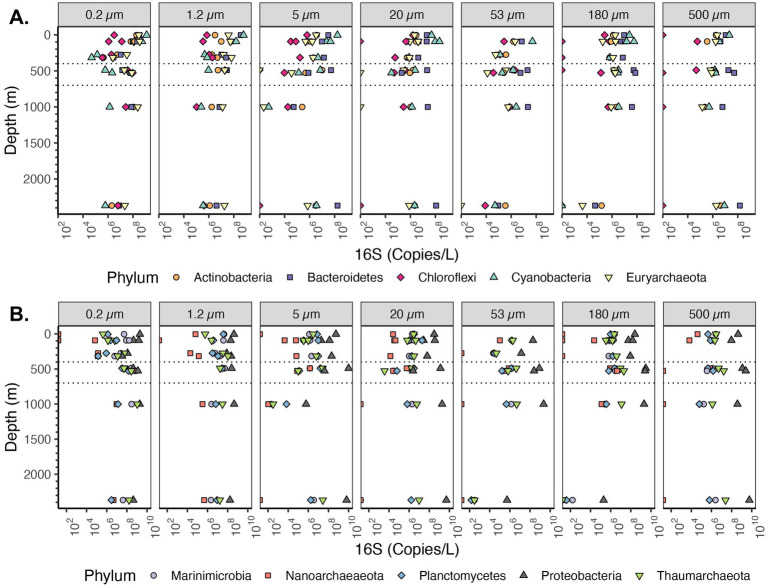
Depth profile of volume normalized 16S rRNA gene copy abundance of top 10 most abundant Phyla in the EPR. The ten most abundant Phyla in the entire dataset are shown. Divided into parts **(A,B)** for easy visualization. Each panel represents different particle sizes (in μm). The Y axis represents depth in meters, and the x axis represents 16S rRNA gene copies per L. The area between the dashed lines corresponds to the Oxygen Deficient Zone.

The most abundant phyla on particles were Bacteroidetes, Cyanobacteria, Planctomycetes and Proteobacteria, but Actinobacteria, Marinimicrobia, Chloroflexi, and Thaumarcheota were also key players in the system that were primarily free-living (Figure 6). We calculated relative microbial abundance (%) and quantitative microbial abundance (in 16S Copies/L). Proteobacteria dominate in the quantitative data at each particle size and across all depths (Figure 6B). The abundance of 16S rRNA gene copy numbers/L in Proteobacteria was up to 10^9^ 16S rRNA gene copies in the larger (>500 μm) particle size, which was similar to values for the free-living fractions (Figure 6B). In relative abundance units, Proteobacteria were still the dominant group (reaching almost 100% in the particles ≥500 μm) and we can see similar changes in Proteobacteria relative abundance as in the quantitative data (Supplementary Figures S3, S4). However, as seen previously (Gloor et al., 2016), the variation of smaller groups appear to be determined by variations in the determined by variations in the dominant group. For example, when examined quantitatively, we can see the changes in the less abundant Planctomycetes phylum across depth in the free-living fraction, including a maximum in the ODZ (Figure 6B). With relative abundance, we lost the information about the presence and variation of Planctomycetes, compared to the most abundant group (Proteobacteria) (Supplementary Figures S3, S4).

The majority of cyanobacteria were from the order Synechococcales, otherwise known as picocyanobacteria (*Synechococcus* and *Prochlorococcus*) (Supplementary Figure S5). The picocyanobacteria are generally free-living, but known to attach to particles under specific conditions (Capovilla et al., 2023). Abundances of Cyanobacteria, which use light for photosynthesis, changed sharply with depth. In the euphotic zone, Cyanobacteria were the second most abundant phyla on particles for almost all size fractions (Figure 6A). For the free-living fractions 0.2 and 1.2–5 μm particles, Cyanobacteria decreased from 10^9^ 16S rRNA gene copies/L in the surface to 10^6^ 16S rRNA gene copies/L in the mesopelagic region. For particles of size 5–20 μm, the decrease was almost linear, changing from 10^8^ 16S rRNA gene copies/L in the surface to 10^3^ in the mesopelagic. In the size fractions 20–53 μm and 53–180 μm, Cyanobacteria were 10^8^ 16S rRNA gene copies/L in the euphotic zone while for particles >500 μm Cyanobacteria were 10^7^ 16S rRNA gene copies/L. Thus, though cyanobacteria were abundant on particles, the cyanobacteria were one order of magnitude less abundant on small particles than in the free-living fraction and two orders of magnitude less abundant on large particles.

Among Archaea, the phylum with the highest 16S rRNA gene copies/L corresponded to Thaumarchaeota (Figure 6B). Euryarcheota were more abundant in the <5 μm fractions in the euphotic zone, but otherwise Thaumarchaeota had the highest abundances (Figure 6A). Thaumarchaeota abundance changed with depth and across particle sizes. For example, in the free-living size fraction (0.2–1.2 μm) Thaumarchaeota increased from <10^6^ 16S rRNA gene copies/L in the surface to almost 10^9^ 16S rRNA gene copies/L in the ODZ region and remained high at depth, and a similar pattern was observed in particle size 1.2–5 μm. However, in particle 5–20 μm, the abundance of Thaumarchaeota decreased with depth from ~10^6^ 16S rRNA gene copies/L close to the surface to ~10^5^ 16S copies/L in the ODZ region to 10^2^ 16S rRNA gene copies/L in the bathypelagic region (1,001 m depth). In particle sizes ≥20 μm the Thaumarchaeota abundance mostly remained between 10^6^ and 10^7^ 16S rRNA gene copies/L, including in the bathypelagic region (1,001 m). The abundance of Thaumarchaeota in the non-buoyant plume depth was different at each particle size; it was high 10^7^–10^8^ copies/L in the small size fractions (from 0.2 to 20 μm), but in the 53–180 μm and 180–500 μm size fractions, Thaumarchaeota was <10^2^ copies/L. Finally, on the ≥500 μm size fraction in the non-buoyant plume, there were ~10^7^ 16S rRNA gene copies/L. Thus, while Thaumarchaeota were most abundant in the free-living fraction, they were also present in the majority of particles, but at several orders of magnitude lower abundance.

**Figure 7:**
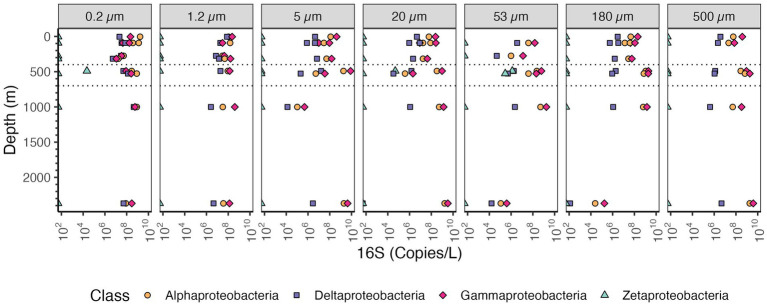
Depth profile of volume normalized 16S rRNA gene copy abundance of Proteobacteria, aggregated to class level in the EPR. The five most abundant classes in the entire dataset are shown. Each panel represents different particle sizes (in μm). The Y axis represents depth in meters, and the x axis represents 16S rRNA gene copies per L. The area between the dashed lines corresponds to the Oxygen Deficient Zone.

**Figure 8:**
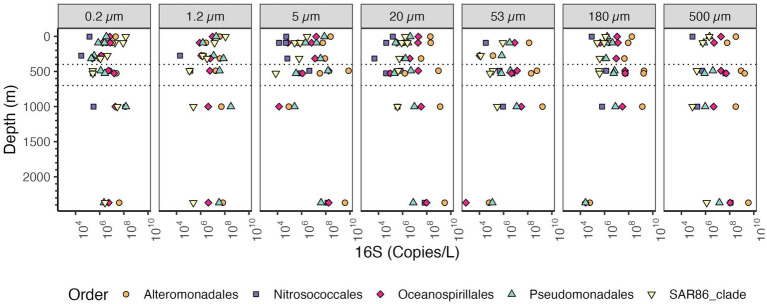
Depth profile of volume and bin size normalized 16S rRNA gene copy abundance of Gammaproteobacteria, aggregated to order level. The five most abundant orders in the entire dataset are shown. Each panel represents different particle sizes (in μm). The Y axis represents depth in meters, and the x axis represents 16S rRNA gene copies per L. Dashed lines indicate the boundaries of the ODZ.

Among the Proteobacteria, the most abundant class in the particle fractions was consistently the Gammaproteobacteria, reaching >10^9^ copies/L in each of the size fractions >20 μm followed by Alphaproteobacteria, reaching 10^9^ copies/L (Figure 7). The most abundant Gammaproteobacterial orders on large particles were Alteromonas (~10^8^ copies/L) and Oceanospiralles (Figure 8) (~10^8^ copies/L). In the free-living fraction Alphaproteobacteria (>10^9^ copies/L) were more abundant than Gammaproteobacteria (10^8^ copies/L) (Figure 7). In the free-living and small particles 1.2–5 μm, the abundance of Alphaproteobacteria varied from 10^7^ to 10^9.5^ copies/L in the water column. In the 5–20 μm size class Alphaproteobacterial abundance decreased almost linearly from the surface ~10^8^ to ~10^5^ copies/L in the Bathypelagic region. In particles in sizes >53 μm, Alphaproteobacteria abundance varied between ~10^7^ to ~10^9^ copies/L, but Alphaproteobacteria abundance increased from the surface to the ODZ. Additionally, Deltaproteobacteria were present throughout the water column, and in all size fractions though at lower abundances than Gammaproteobacteria and Alphaproteobacteria (Figure 7). The abundance of Deltaproteobacteria decreased as particles increased in size; in the free-living and 1.2–5 μm fractions abundance was 10^6^–10^8^ copies/L, but in each of the particle size fractions, abundance was mostly 10^6^ copies/L or less. Members of Zetaproteobacteria were only found in the ODZ and had low numbers (10^4^ copies/L) as free living (0.2–1.2 μm) fraction but were associated with particles 10^5^–10^6^ copies/L on 20–53 and 53–180 μm in the ODZ region (Figure 7).

**Figure 9:**
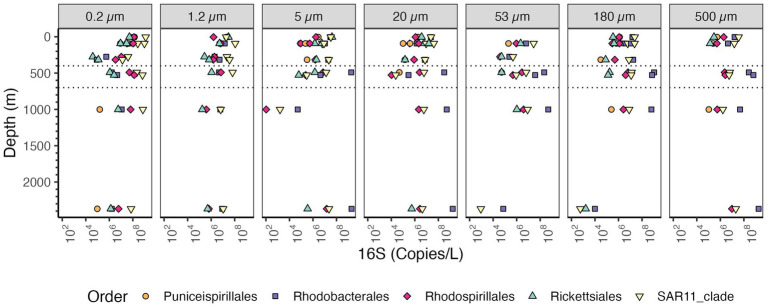
Depth profile of volume normalized 16S rRNA gene copy abundance of Alphaproteobacteria, aggregated to order level in the EPR. The five most abundant orders in the entire dataset are shown. Each panel represents different particle sizes (in μm) The Y axis represents depth in meters, and the x axis represents 16S rRNA gene copies per L. Dashed lines indicate the boundaries of the ODZ.

SAR11 and Rhodobacterales were the most abundant Alphaproteobacteria orders present on all sizes of particles (Figure 9). Rhodobacterales clearly increased in abundance in the free-living fraction in the ODZ as did Rhodospirillales and SAR11. Rhodobacterales were the most abundant Alphaproteobacteria on >5 μm particles in the ODZ and below while SAR11 was the most abundant Alphaproteobacteria on particles in the top 300 m. SAR11 bacteria are an order of Alphaproteobacteria that are thought to be free-living (Giovannoni, 2017). In general, the total SAR11 abundance was higher in the free-living size class (0.2–1.2 μm) where the abundances of SAR11 decreased from ~10^9^ 16S rRNA gene copies/L in the surface to 10^6^ 16S rRNA gene copies in the mesopelagic oxycline above the ODZ, but in the ODZ SAR11 increased to ~10^8^ 16S rRNA gene copies/L, and remained relatively constant at deeper depths. On larger particles, the abundance of SAR11 varied between ~10^7^ to 10^8^ 16S rRNA gene copies/L (Figure 9). Within SAR11, we detected four clades (Clades I, II, III, and IV) as well as Unclassified SAR11 that did not fall into any clade (Supplementary Figure S6). The clade that had the highest abundances both in the free-living and particle fractions was Clade I, closely followed by Clade II. Both clades were found at almost every analyzed depth. The abundance of all SAR11 clades decreased as particle size increased (Supplementary Figure S6). Thus, there was no significant shift in clades between the water and particles. All these clades break down into smaller ecotypes, which is the more appropriate level to examine for environmental distributions (Haro-Moreno et al., 2020), but which we cannot resolve with these methods.

**Figure 10:**
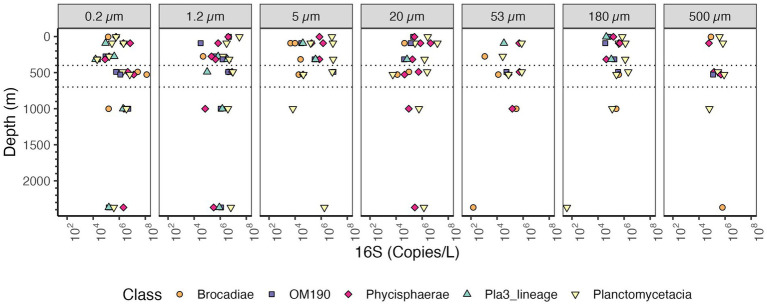
Depth profile of volume normalized 16S rRNA gene copy abundance of Planctomycetes, aggregated to Class level. The five most abundant classes are shown for the entire dataset. Each panel represents different particle sizes (in μm). The Y axis represents depth in meters, and the x axis represents 16S rRNA gene copies per L. The area between the dashed lines corresponds to the Oxygen Deficient Zone.

The majority of taxa from the Planctomycetes phylum are known particle attached bacteria except for anammox bacteria which are free-living in marine ODZs (Fuchsman et al., 2012). Examination of the Planctomycetes phylum as a whole, indicates that the samples with the highest Planctomycetes were the free-living fractions in the ODZ region ~10^8^ copies/L (Figure 6A). In the surface waters (0.2–1.2 μm), when examined in comparison to Proteobacteria >10^9^ copies/L, there were few Planctomycetes 10^6^–10^7^ copies/L. At non-ODZ depths, Planctomycetes remained at 10^6^–10^7^ copies/L in each of the particle size fractions 20 μm and above while Proteobacteria decreased to ~10^8^ copies/L (Figure 6A). Thus, in terms of relative abundance, Planctomycetes were enriched in particles (Fig. S3 and S4), but in actual abundances, they were similar between free-living and suspended particles (Figure 6). We looked into the abundance of classes inside Planctomycetes. Planctomycetes of the group Brocadia, which mediate the anammox reaction, were highly abundant (~10^8^ copies/L) in the free-living fraction in the ODZ (Figure 10), which is consistent with previous data (Fuchsman et al., 2017, 2012; Ganesh et al., 2014). The class Planctomycetacia, which includes Pirellulaceae and Planctomycetales orders among others, had the highest abundance across particle sizes among the Planctomycetes, with 10^6^–10^7^ 16S rRNA gene copies/L in each fraction (Figure 10). However, in the ≥500 μm fraction the abundance of Planctomycetacia decreased to ~10^6^ 16S rRNA gene copies/L (Figure 10).

Bacteroidetes, particularly Flavobacteria, is a second group that is generally considered particle attached (Delong et al., 1993; Eloe et al., 2011; Fuchsman et al., 2011; LeCleir et al., 2014; Leu et al., 2022). In the EPR, Bacteroidetes was the second most abundant phyla on large particles below the euphotic zone, after Proteobacteria. Our data indicate that in the free-living fraction the Bacteroidetes group was more prevalent in the euphotic zone than at depth. However, in the large particles, Bacteroidetes copies were similar in the euphotic zone and at depth. We observed that Bacteriodetes had a higher number in small particle fractions with 10^8^ copies/L in both free-living and < 20 μm particles but 10^7^–10^8^ copies/L in particle fractions >20 μm (Figure 6A). Thus, the pattern was fairly similar between Planctomycetes and Bacteroidetes, both of which are generally considered particle attached.

We also examined the abundance of nitrite-oxidizing *Nitrospina* in our samples due to its ecological relevance in the nitrogen cycle. *Nitrospina* abundance was higher in the free-living fraction (0.2–1.2 μm) specially inside the ODZ region, where *Nitrospina* abundance increased to ~10^8^ copies/L (Supplementary Figure S7). In the surface and in the non-buoyant plume the abundance was one order of magnitude lower ~10^6^ copies/L. The abundance of *Nitrospina* decreased one order of magnitude in the larger particle sizes, at 1.2–5 μm the highest abundance was in the ODZ with ~10^6^ copies/L, and in the larger particle size (≥20 μm) the *Nitrospina* abundance decreased to ~10^5^–10^6^ 16S rRNA gene copies/L. Thus, *Nitrospina* was primarily free-living.

**Figure 11:**
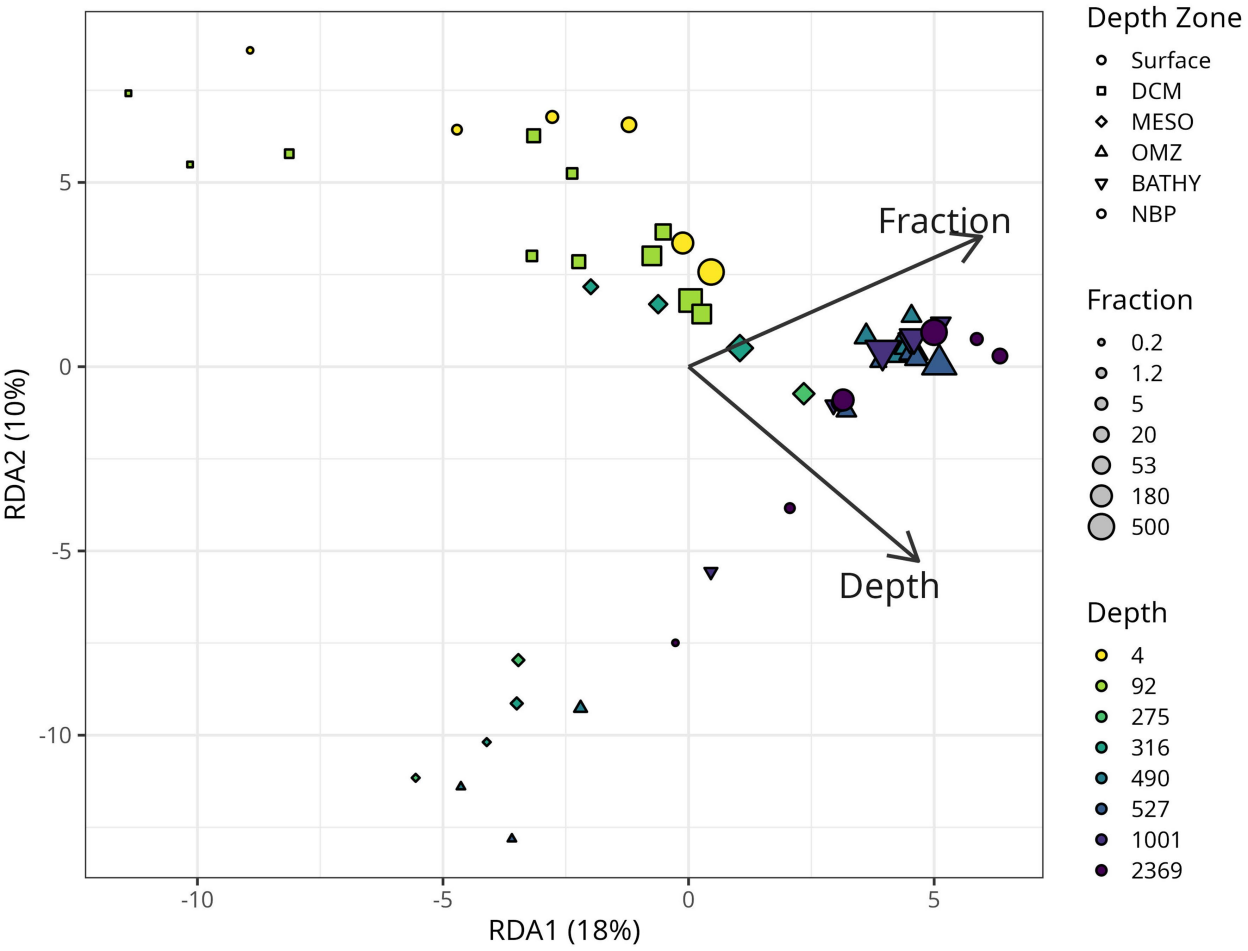
Redundancy analysis plot (RDA) of the relationship between microbial community structure, particle size “fraction” and depth. Points that are closer together in space have more similar community structure. The RDA model was fitted using log-transformed values of depth and fraction, including quadratic and interaction terms, to capture nonlinearities and synergistic effects. For clarity, however, the biplot displays arrows labeled simply as depth and fraction, which represent the main-effect coefficients derived from the full model. Arrow lengths indicate the strength of the relationships, while their directions show the gradients along which community composition shifts. Although the plotted coefficients are not log-transformed, all analyses were conducted on log-transformed data, and the higher-order terms were statistically significant (*p* < 0.01). The first two axes explain 18 and 11% of the total sample variance.

Redundancy Analysis (RDA) comparing taxonomic diversity against depth and size fraction generally suggested that large particles (≥180 μm) appear to be more similar to each other, regardless of location, and that smaller particles appeared to be more dissimilar between depth regions (Figure 11). We observed significant nonlinear (quadratic) relationships of both depth (*p* = 0.005) and size fraction (p = 0.005), as well as a significant interaction between these variables (*p* = 0.001), in shaping community structure.

## Discussion

The analysis of microbial amplicon sequences has been limited by the use of relative abundance as an indicator of microbial diversity. Here we use internal spike-in standards to quantitatively address how microbial composition and organic C change among seven particle size fractions in a depth profile of the open ocean water column located above the 9°50’N East Pacific Rise hydrothermal vent field. The 9°50’N EPR region examined here had a defined low oxygen zone where oxygen was below detection (400–700 m depth), and in the deepest region sampled reached a non-buoyant plume (2,369 m depth) sourced from a hydrothermal vent.

### Ecology of sinking particles in the EPR

Our data showed a statistically significant, though non-linear relationship between depth and size fraction and the microbial community structure (Figure 11), reflecting previous findings using related methods in the global ocean (Mestre et al., 2018) and in the Chesapeake Bay (Cram et al., 2024). Together these data suggest that microbial communities have biogeographic ranges that are defined not only at the macro-scale across depth and space, but also at the microscale between different sizes of particles. There was a large increase in the bacterial 16S rRNA copies/ug C and copies/L on particles 180–500 μm and >500 μm between the upper water column and the deep water column (Figure 3). This implies that, even though the total concentration of organic C in particles decreased in the deep water column, the density of bacteria on large particles increased at depth.

In our dataset, the most abundant bacterial phyla on particles were Bacteroidetes, Cyanobacteria, Planctomycetes, Gammaproteobacteria and Alphaproteobacteria (Figures 6, 7). These results are generally comparable to those found previously, with some key exceptions. Campylobacterota have mismatches with the primers used (McNichol et al., 2021), and were barely present in this dataset. This was unfortunate since Campylobacterota (*Arcobacter*), and Alteromonadales Gammaproteobacteria dominated metagenomes from sinking particles at 4000 m depth at HOT (Boeuf et al., 2019). Additionally, Campylobacterota (Sulfurovumaceae and Sulfuricurvaceae) have been found to be particularly enriched in sinking particles from hydrothermal vent plumes sampled 30 m and 115 m from the closest vent chimneys (Sylvan et al., 2012). In our 2,369 m depth particles, Campylobacterota, were <10^4^ copies/L, but Alteromonadales were present in high numbers at >10^9^ copies/L as expected (Figure 8). Thus we illustrate that, even when using well established primer sets regularly used in the marine environment, e.g., (Reintjes et al., 2023; Yeh and Fuhrman, 2022), PCR bias causes environmentally important groups to be missed.

### Considering traditional free-living and particle attached bacteria quantitatively

Marine particles were inhabited by both traditionally particle attached bacteria like Planctomycetes and Bacteroidetes, as well as by free-living SAR11, Thaumarchaeota and picocyanobacteria. In surface waters, where Thaumarchaeota are typically not abundant (Santoro et al., 2017), numbers were similar between Thaumarchaeota on particles and in the water column. At depth, Thaumarchaeota were present in >5 μm particles, but they were an order of magnitude more abundant in the free-living and 1.2–5 μm fractions. 16S rRNA copies of SAR11 bacteria were abundant in the free-living fraction (0.2–1.2 μm), and were also present in larger particle sizes, but in lower abundances (Figure 9). SAR11 has been previously described as a free-living organism (Giovannoni, 2017), and it dominates free-living size fractions, e.g., (Reintjes et al., 2023). This is in concordance with the high abundances in the free-living fraction we observed in our samples (Figure 9). However, in the top 300 m, SAR11 was also often the most abundant Alphaproteobacteria on particles (Figure 9). Despite SAR11’s presence on particles, it was still two orders of magnitude lower in abundance than in the free-living fraction. Several other papers with size fractionated amplicon data have found SAR11 on particles (Cram et al., 2024; Mestre et al., 2020; Yeh and Fuhrman, 2022). However, it is possible that abundant free-living microbes are entrained in particles as a result of viral infection (Fuchsman et al., 2019a), grazing and defecation, or physical aggregation processes. Previous work indicated that the surface Thaumarcheota ecotypes were the main ecotype found in deep particles (Ijichi et al., 2019), which is consistent with entrainment of free-living microbes into particles in the euphotic zone. The equal numbers of Thaumarcheota on particles and free-living in surface waters is harder to explain. It is also possible that free-living microbes were entrained into the particle fractions while sampling, but as seen in the methods, these samples were gravity filtered and had no clogging. SAR11 bacteria have evolved a particularly non-sticky less hydrophobic cell surface to avoid predation (Dadon-Pilosof et al., 2017). Thus, SAR11 bacteria should be less likely to stick to filters than other bacteria, so we suspect that their presence on particles is not an artifact.

Planctomycetes are considered a particle attached group (Delong et al., 1993; Eloe et al., 2011; Fuchsman et al., 2012; Ganesh et al., 2014; LeCleir et al., 2014; Leu et al., 2022). The abundance of Planctomycetes in particles, could be explained by their ecological role as they respire complex organic matter such as polysaccharides (Sun et al., 2021). The highest abundance of Planctomycetes 16S rRNA was actually found in the free-living fractions in the ODZ (~10^8^ copies/L) due to autotrophic anammox bacteria (Brocadia; Figure 10). At non-ODZ depths, there were similar numbers of Planctomycetes 16S rRNA copies in the free-living fractions and in each particle fraction (Figure 6A). However, if the particle fractions are added together, more Planctomycetes 16S rRNA was present on particles than in the water column. So, in our analysis, Planctomycetes are still a particle attached group, but are also found in the water column in higher abundances than previously thought.

We can also examine Bacteroidetes, a second group that is generally considered particle attached, (Delong et al., 1993; Eloe et al., 2011; Fuchsman et al., 2011; LeCleir et al., 2014; Leu et al., 2022). We observed that Bacteroidetes 16S rRNA had a higher abundance in small particle fractions (< 20 μm particles) and in the free-living fraction, with ~10^8^ copies/L in both, but <10^7^ copies/L in particle fractions >20 μm. Thus, the pattern was fairly similar between Planctomycetes and Bacteroidetes, both of which are generally considered particle attached. These data, in which particle associated organisms are actually similarly abundant in the free-living phase, cause us to rethink the conventional understanding of the habitats of “particle attached” organisms.

We can see that, integrated across all particles, Planctomycetes and Bacteroidetes are more abundant in particles than in the water column. However, their abundance in the free-living phase has previously been overlooked. The significant presence of particle attached bacteria in the free living phase is consistent with the published models that assume that particle attached bacteria attach to a particle and then detach again as the particle sinks (Nguyen et al., 2022). In this model, microbes are constantly colonizing particles as they sink rather than having colonization only at the surface, allowing the microbes to operate in the pressure zone where they are adapted (Nguyen et al., 2022). Microbial lifestyles complicate, but are generally consistent with, this picture. For example, many Planctomycetes have a sessile attached life phase that produces swimming swarmer daughter cells (Fuerst and Sagulenko, 2011). In this case, the Planctomycetes in the free-living and particle attached fractions may be in different life stages. We do know that some particle attached microbes, such as Bacteroidetes and Planctomycetes, are transported to the bathypelagic ocean during high flux pulses (Poff et al., 2021), but these microbes may not thrive there. About 10% of the bathypelagic microbes (1000–4,000 m) have reduced activity under high pressure, including Bacteroidetes and *Alteromonas* (Amano et al., 2022). It is the free-living reservoir of particle attached microbes that allows attaching to particles to be a legitimate evolutionary strategy.

### Microbial communities in the ODZ

In the EPR, the Seabird oxygen sensor on the CTD reported oxygen concentrations of 2.6 μM from 450 to 850 m. Standard CTD oxygen sensors are unable to differentiate very low oxygen concentrations and oxygen concentration that are at a minimum value but unchanging over depth have previously been used to identify anoxic water (Kwiecinski and Babbin, 2021), suggesting that oxygen concentrations were likely less than the detection threshold of the CTD sensor. Additionally, this same region harbored a N deficit and a secondary nitrite maximum, suggesting the presence of denitrification (Figure 2). The secondary nitrite maximum was only 0.3 μM, which is close to but below the threshold of 0.5 μM suggested to confirm anoxia (Banse et al., 2017). However, our nutrient sampling was sparse with 1 point in the ODZ, so depths with higher concentrations could easily have been missed. The N deficit was in the same concentration range as previously seen in the core of the ETNP ODZ (Fuchsman et al., 2018), and we cannot prove that the this signal was produced *in situ* in the EPR region rather than advected from the ETNP core. With this chemical dataset, we cannot determine the difference between suboxia and anoxia. However, the microbial community can give us insight in this manner. The high abundance of Thaumarchaeota (ammonia oxidizing archaea) and the presence of nitrite oxidizing bacteria *Nitrospina* in the ODZ (Figure 6 and Supplementary Figure S7), implies that small amounts of oxygen were likely still present. While *Nitrospina* can use oxygen at <4 nM, Thaumarcheota archaea have a higher oxygen threshold (K_m_ = 333 nM O_2_) (Bristow et al., 2016). In the core of the truly anoxic ETNP ODZ, Thaumarcheota and *Nitrospina* were not present (Fuchsman et al., 2017). On the other hand, anammox bacteria, which are 50% inhibited at 880 nm O_2_ (Dalsgaard et al., 2014), were also present at this EPR station. The ecosystem in the EPR ODZ may be similar to that of the extremely hypoxic Bay of Bengal, with oxidative N processes dominating in the water column but anammox still present (Bristow et al., 2017).

Bacterial and archaeal 16S rRNA abundance increased in the ODZ region in the free-living fraction, and in fractions >20 μm (Figure 5). The 16S rRNA abundance in the 5–20 μm fraction did not increase in the ODZ. Rather, abundances had a linear decrease from ~10^8^ copies/μg org C at the surface towards 10^6^ copies/μg org C for bacteria at 1000 m depth (Figure 4). However, on larger particle sizes (≥20 μm) we observed a decrease in abundance from the surface to the low oxygen mesopelagic water above the ODZ, but in the ODZ, (400–700 m depth), the abundance increased in all fractions ≥20 μm, reaching ~10^9^ copies/μg org C. Assuming the EPR is representative of ODZs, reduced flux attenuation in ODZs (Keil et al., 2016; Van Mooy et al., 2002) does not appear to be due to the presence of fewer microbes on particles.

We can examine which microbes are causing this increase in 16S rRNA abundance In the ODZ region. In the free-living fraction, Thaumarcheota, Marinimicrobia, Actinobacteria, *Nitrospina* and Chloroflexi were all abundant (Supplementary Figure S9). One of the groups that only appears in the ODZ were Zetaproteobacteria (Figure 7). Although present in low numbers in the ODZ, Zetaproteobacteria were almost absent in the oxic part of the water column. Known Zetaproteobacteria are all autotrophic iron oxidizers, generally found at hydrothermal vents and in the sediments (McAllister et al., 2019). Reduced iron is often associated with ODZs (Kondo and Moffett, 2013; Vedamati et al., 2014). Additionally, some other groups like Planctomycetes and *Nitrospina* had unusually high abundances in the ODZ water column (Figure 6 and Supplementary Figure S7 respectively). Brocadia, or anammox bacteria, were the most abundant Planctomycetes in the ODZ though Planctomycetacia and Phycisphaera were also abundant (Figure 10). Anammox bacteria are known to primarily reside in suboxic and anoxic waters (Dalsgaard et al., 2014). Inside the ODZ region, the nitrite oxidizer *Nitrospina* had high abundance in the free-living fraction with ~10^7^ -10^8^ copies/L (Supplementary Figure S7). This increase in *Nitrospina* abundance can be related to the presence of nitrite in the ODZ (Figure 2B). Additionally, more ubiquitous bacteria such as SAR11, which have specific ODZ ecotypes (Tsementzi et al., 2016), also had a maximum in the ODZ (Figure 9).

Previous work has also found increases in particle number (Cram et al., 2022), particle flux (Fuchsman et al., 2019a) and cell counts (Ulloa et al., 2012) in ODZ regions. Potential reasons hypothesized for these increases include higher rates of chemosynthesis, photosynthesis when ODZs are at shallower depths than seen here and active transport by zooplankton who migrate into ODZs to avoid predation (Cram et al., 2022; Fuchsman et al., 2019a; Ulloa et al., 2012).

### The non-buoyant plume (NBP) had an unique microbial community structure

Although our sampling did not reach the ocean floor, where the hydrothermal vents are located in the EPR, we analyzed a sample at 2369 m depth, 163 m from the bottom, which was characterized using beam transmission as containing particles from the hydrothermal vent’s non-buoyant plume (Supplementary Figure S2). This sample had a unique structure, where the 16S rRNA abundance did not follow the trends or patterns we observed through the water column at shallower depths. For example for the free living fraction the 16S rRNA abundance of bacteria in the Non-Buoyant plume region was similar to the numbers in the ODZ (~10^9^ copies/L; Figure 5), but for the 53–180 and 180–500 μm size fractions the abundance was ~10^9^ copies/L in the ODZ, but 10^6^ copies/L at the Non-Buoyant plume. In the 53–180 and 180–500 μm size fractions, many of the typical bacteria, such as Planctomycetes, were missing and the number of Proteobacteria 16S rRNA copies were greatly decreased (Figure 6B, Supplementary Figure S9). In these size fractions, Pseudomonodales, rather than Alteromonadales, were the dominant Gammaproteobacteria (Figure 8). Non-Buoyant plumes evolve as they move away from the hydrothermal vent, with fewer vent related Campylobacterota and more SAR11, S-oxidizing *Thioglobus* Gammaproteobacteria and S-oxidizing *Sulfurimonas* Campylobacterota farther from the vent (Li et al., 2020). In our samples, in the Non-Buoyant plume, we found *Thioglobus* Gammaproteobacteria (Supplementary Figure S8), but Campylobacterota were almost absent in all of our samples, likely due to PCR biases. We observed SAR11 in our free-living non-buoyant plume sample (2,369 m depth) in high abundance (10^8^ copies/L), but it was much less abundant in in 53–180 and 180–500 μm plume particle samples (10^3^ copies/L) (Figure 9). The phylum Acidobacteria was also abundant in the free-living plume samples, but was not abundant at other depths (Supplementary Figure S9).

### Methodological advances and caveats

This work includes three main technological advances. First, the use of 16S rRNA gene sequence spike-in allowed us to look quantitatively into microbial abundance across different particle sizes at different ocean depth ranges. This approach allowed us to compare microbial abundance of less abundant taxa directly across particle samples, rather than using relative abundance which is greatly affected by the abundance of the most abundant microbes. Secondly, we describe the microbial diversity and abundance in marine particles separated according to seven size-fractions from 0.2 to > 500 μm. This is many more particle sizes compared to other studies in ODZs that have only focused into 2 size fractions: 0.2–1.6 μm and >1.6 μm (Ganesh et al., 2014) or 0.2–30 μm and >30 μm (Fuchsman et al., 2017). This work has allowed us to examine how microbial communities change across the full particle size spectrum, and consider small, intermediate and large particles separately. Thirdly, we also examine microbial abundance quantitatively in relation to organic matter concentrations.

However, there are several caveats to this work. We learned, based on the non-detection of spike in some samples, and the low detection of spike in others that 10^4^ spikes per ng of DNA is not enough spike copies. We therefore recommend using at least 10^5^ spikes per ng of environmental DNA if using an illumina MiSeq or similar instrumentation. Greater sequence depth may allow use of similar or lower spike abundance. All PCR based environmental sequencing approaches including this one are subject to amplification biases which under-represent some species and over-represent others (Elbrecht and Leese, 2015). For example, the primers used here are known to not amplify Campylobacterota (McNichol et al., 2021), which are important in particle systems (Boeuf et al., 2019; Leu et al., 2022). Surprisingly, while McNichol et al. (2021) identified a primer mismatch to the order *Brocadiales,* which include anammox bacteria, we identified many *Brocadiales* bacteria in this dataset (Figure 10), suggesting that the mismatch may still allow amplification of this group. However, such a mismatch might lead to under amplification – it is possible that *Brocadiales* are even more abundant than indicated herein. Additionally, 16S rRNA gene corresponds to identity not function; in previous particle metagenomes, there were found several genes involved in denitrification (*nosZ, nirK, qnorB*) enriched in particles (Fuchsman et al., 2017; Ganesh et al., 2014). However, denitrifiers are phylogenetically diverse and it is very difficult to identify them based on 16S rRNA gene sequences (Bertagnolli et al., 2020; Zhang et al., 2023; Zumft, 1997). Another confounding factor is that the 16S rRNA gene is not a single copy core gene. Many bacteria contain multiple copies of the 16S rRNA gene (1–15) (Espejo and Plaza, 2018; Venter et al., 2004). Multiple gene copies per cell would cause our gene copy counts to be higher than true microbial cellular abundances. Indeed in the Chesapeake Bay, the amplicon-based estimates of microbial abundance were generally higher than microscopy counts from the particles (Cram et al., 2024). We advocate similar quantitative and size fractionated work with metagenomes, which would eliminate PCR amplification biases, allow the examination of functional genes for denitrification and sulfate reduction, and would allow the use of known single copy core genes for microbial enumeration. Despite these limitations, the patterns in 16S rRNA gene abundances here should reflect the patterns in their host organisms, allowing us to examine patterns in microbial abundance across a full size spectrum and in relation to organic C in the open ocean for the first time.

## Concluding remarks

Combining 16S rRNA gene standard spike in amplicon sequencing and fractionation of particles at a finer scale allowed us to look into the diversity and abundance of free-living and particle-attached microbes from a new perspective. When we compared the quantitative abundance of different microbial taxa across depths and particle sizes, we observed increases in abundance of many groups in the ODZ region and in the non-buoyant hydrothermal vent plume, both regions that promote chemosynthesis.

There was a large increase in the bacterial 16S rRNA copies/ug C and copies/L on particles >180 μm between the upper water column and the deep water column. We expect that the density of bacteria on large particles was increasing with depth. Quantitative abundance estimates found that ostensibly particle attached bacteria such as Bacteroidetes and Planctomycetes were most abundant on particles, but still had many members in the free living state. Such patterns would not have been evident if we had looked at relative abundance data only, where the changes in total numbers of microbes between the water and particles are not taken into account. The significant presence of particle attached bacteria in the free living phase is consistent with the published models that assume that microbes are constantly colonizing and detaching from particles as they sink, allowing the microbes to operate in the pressure zone where they are adapted (Nguyen et al., 2022).

Indeed, models of microorganisms on particles have provided a valuable description of the microbial attachment, detachment and growth dynamics on particles (Mislan et al., 2014; Nguyen et al., 2022; Stephens et al., 2024). However, these models are limited by existing data in two ways. First, they have either not included observations of microbial diversity (Mislan et al., 2014; Nguyen et al., 2022) or else compared their model results to relative abundances of observed marine microbes on sinking particles (Stephens et al., 2024). Secondly, considering the continuum of particle sizes provides valuable insights into microbial processes (Cram et al., 2022, 2018; Leung et al., 2021; Weber and Bianchi, 2020) but microbially resolved models have not to date considered the full size spectrum. In fact, this work is the first time microbial abundance and particle organic C concentrations have been measured together across the size spectrum in the ocean. These data pave the way for more quantitative models of microbial processes on particles of a range of sizes.

## Data Availability

The sequence data are available in NCBI’s Short read archive under BioProject PRJNA1191024. CTD data are in the R2R Rolling deck repository (https://www.rvdata.us/search/cruise/AT42-09). Nutrient data have been deposited in BCO-DMO (doi: 10.26008/1912/bco-dmo.948718.1). Organic C concentrations have been deposited in BCO-DMO (doi: 10.26008/1912/bco-dmo.948709.1). A table with the abundances of each ASV has been deposited at FigShare (doi: 10.6084/m9.figshare.28887038.v1).
