# Supplementary material for: Quantitative microbial taxonomy across particle size, depth, and oxygen concentration
Source: Front Microbiol. 2025 May 23;16:1552305. doi: 10.3389/fmicb.2025.1552305 (PMC12142689; doi:10.3389/fmicb.2025.1552305)
Supplement: Supplementary file 1 [file Supplementary_file_1.zip › Supplementary Figures.PDF]

Supplementary Figures for "Quantitative microbial taxonomy across particle size, depth, and oxygen concentration" by Paulina Huanca-Valenzuela, Clara A. Fuchsman, Benjamin J. Tully, Jason B. Sylvan, and Jacob A. Cram

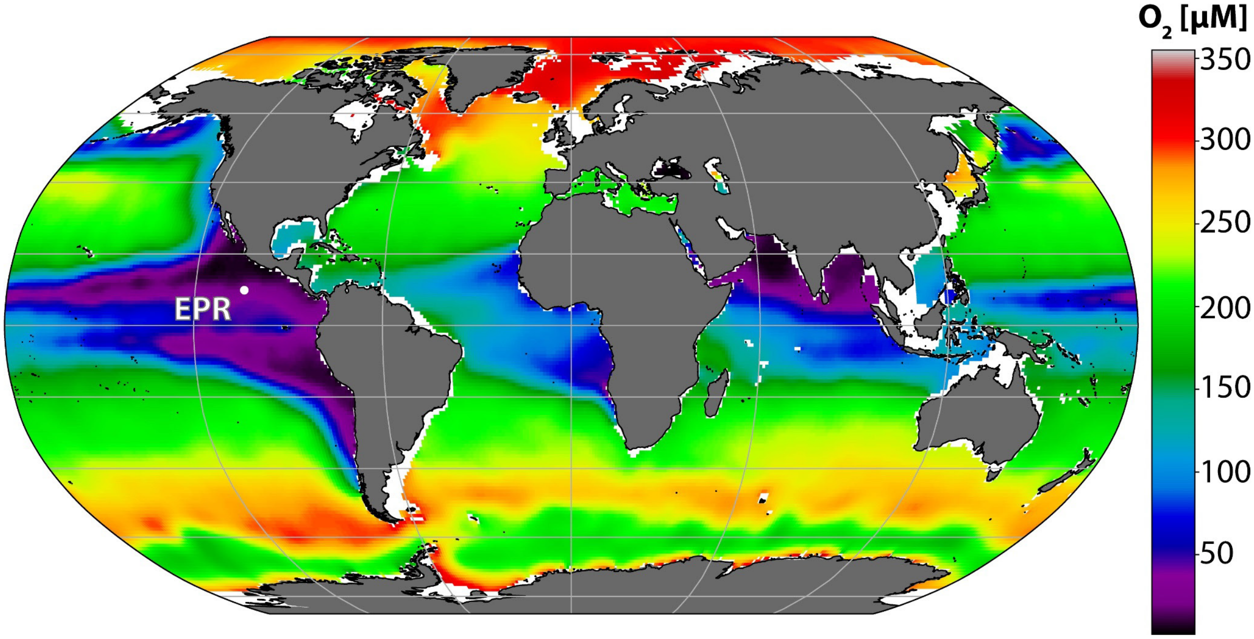

Supplementary Figure 1. Map of Oxygen concentration at 300 m using data from World Ocean Atlas 2018 (Garcia et al., 2019). Location of the EPR station indicated by the white dot.

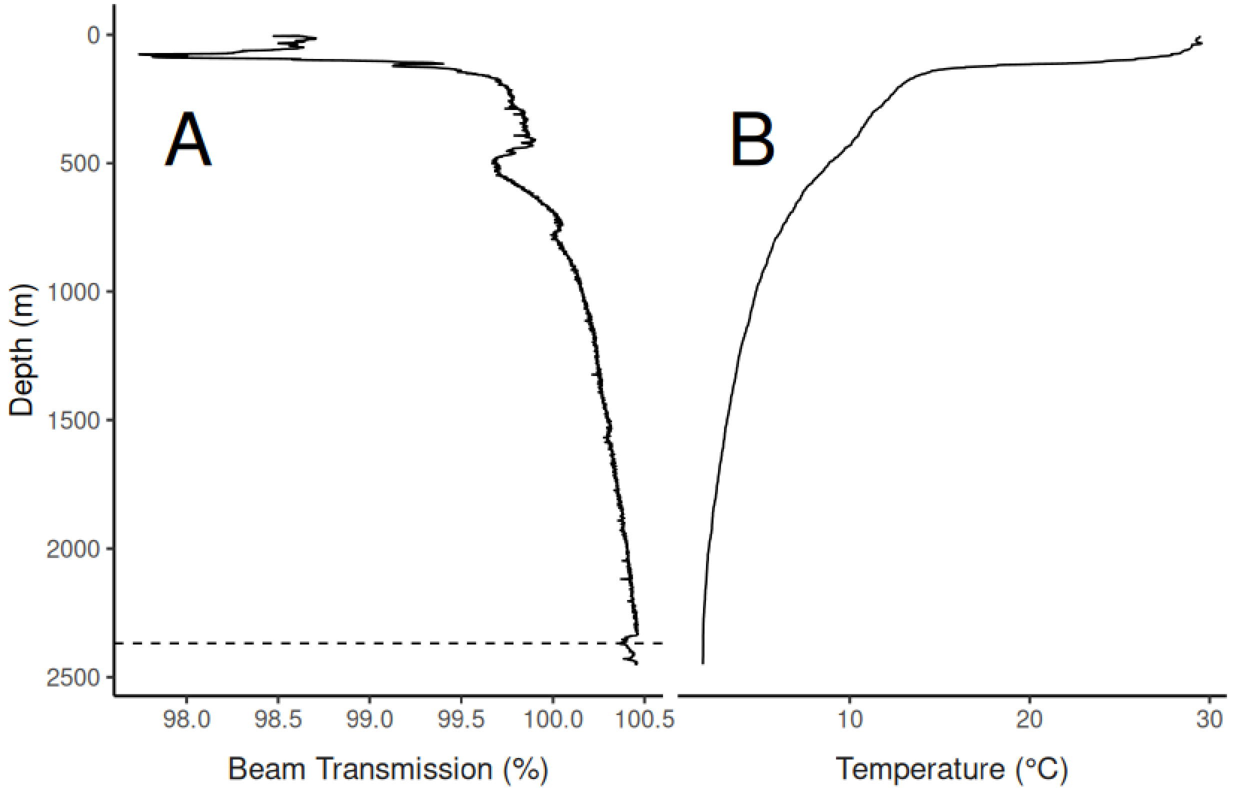

Supplemental Figure 2. A) Beam transmission and B) temperature profiles. The dashed horizontal line indicates the depth of sampling for the non-buoyant plume sample. This is the same cast from which DNA for the plume was sampled. Sampling depth is indicated by a dashed line.

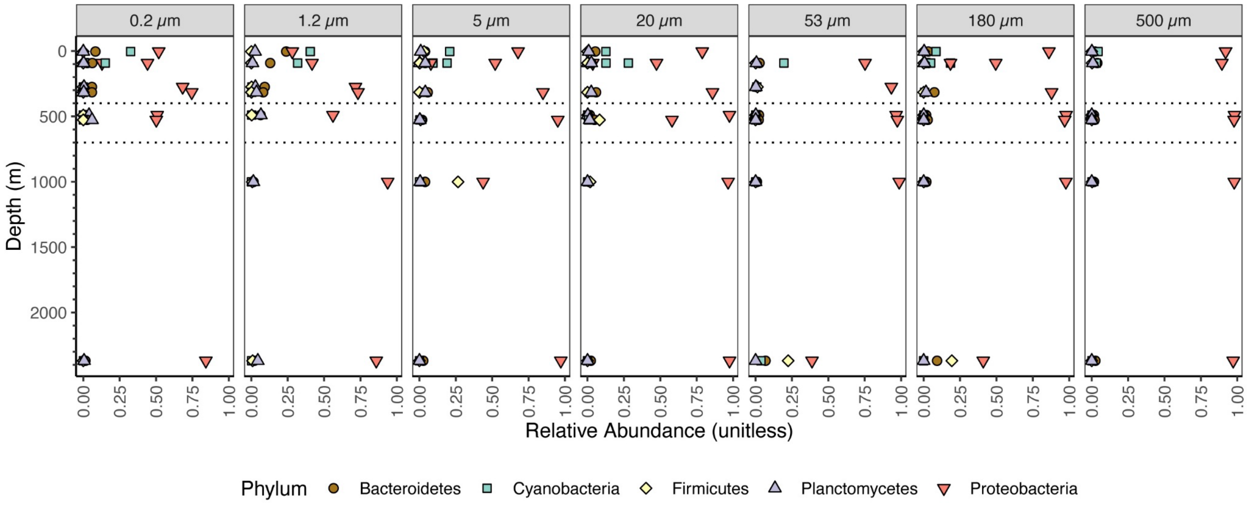

Supplementary Figure 3. Relative abundance of Bacterial 16S rRNA gene copy numbers aggregated to the Phylum level. The five most abundant phyla for the entire dataset are shown. The area between the dash lines corresponds to the Oxygen Deficient Zone.

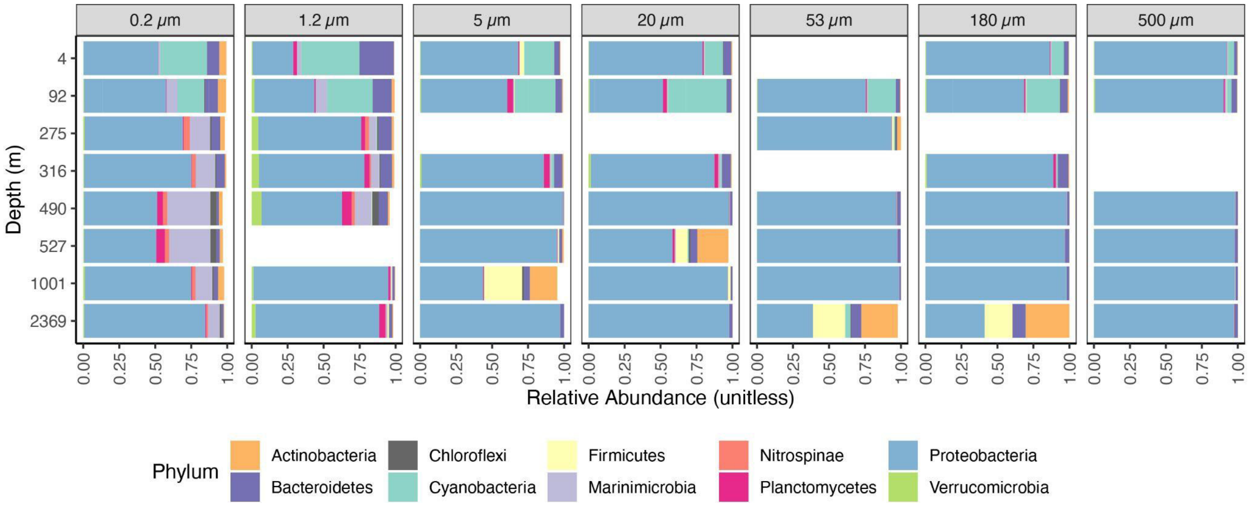

Supplementary Figure 4. Stacked bar chart showing relative abundance of bacterial 16S rRNA genes aggregated to the Phylum level. The ten most abundant phyla are shown for the entire dataset. The area between the dash lines corresponds to the Oxygen Deficient Zone. The depth is not to scale.

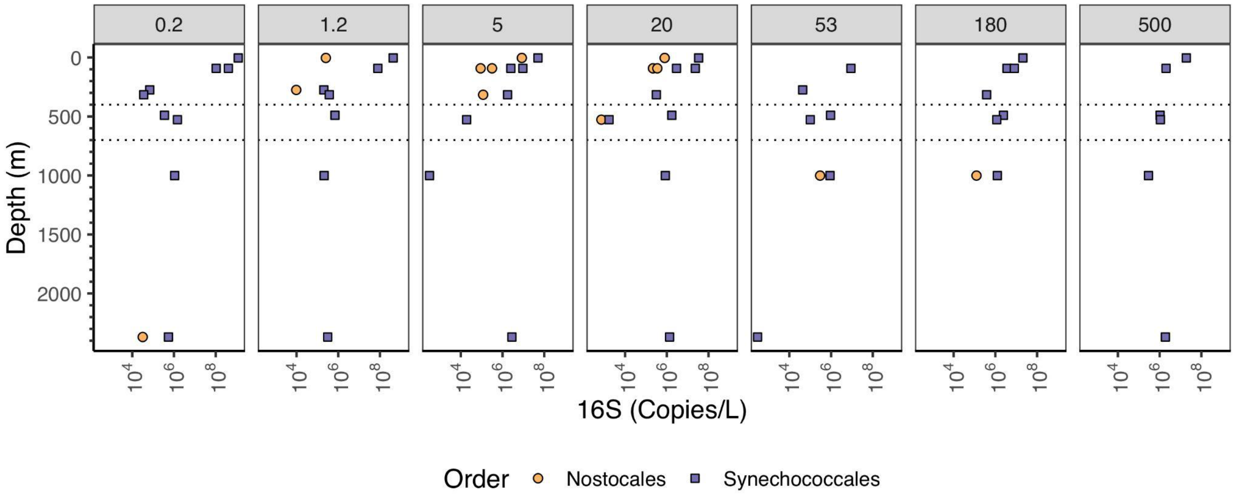

Supplementary Figure 5. Abundance of 16S rRNA gene copy numbers of Cyanobacteria aggregated to order level**.** Each panel represents different particle sizes (in µm). The Y axis represents depth in meters, and the x axis represents 16S rRNA gene copies per L. The area between the dash lines corresponds to the Oxygen Deficient Zone.

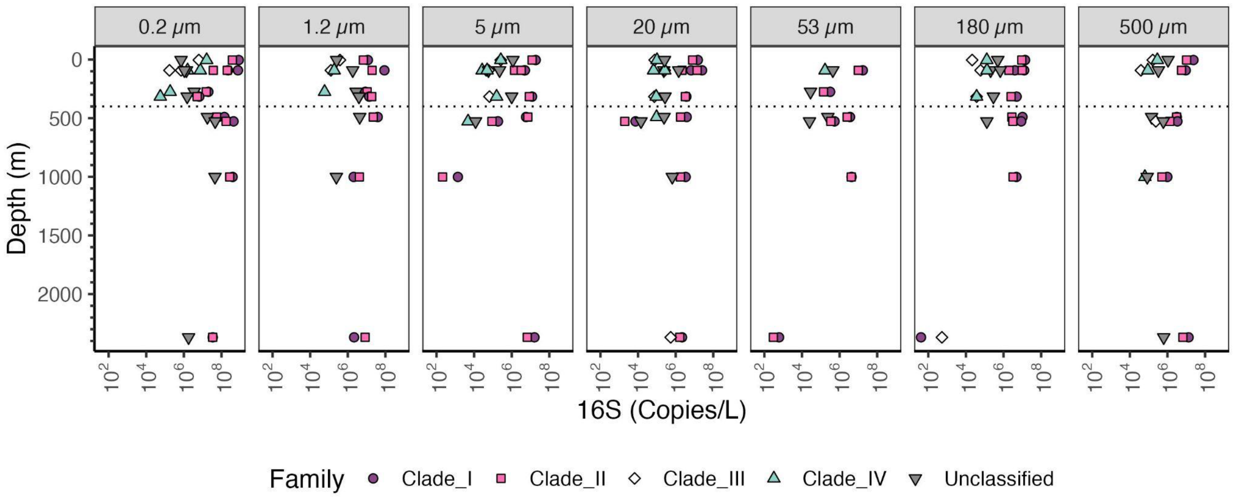

Supplementary Figure 6. Depth profile of volume and bin size normalized 16S rRNA gene copy abundance of SAR11, aggregated to Family level. Each panel represents different particle sizes (in μm). The Y axis represents depth in meters, and the x axis represents 16S rRNA gene copies per L. The area between the dashed lines corresponds to the Oxygen Deficient Zone.

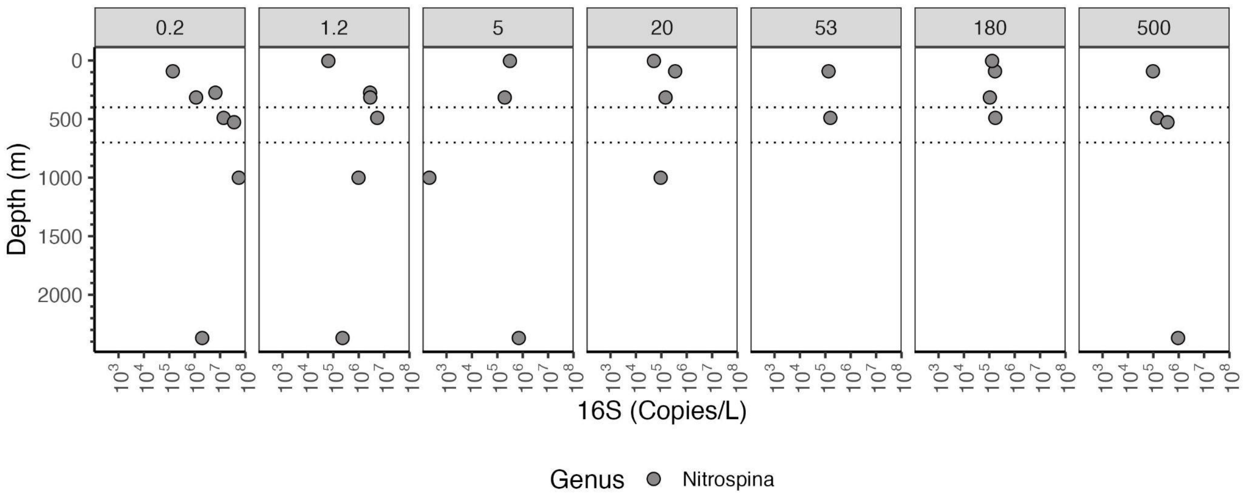

Supplementary Figure 7. Abundance of 16S gene copy numbers of *Nitrospina.* Each panel represents different particle sizes (in μm). The Y axis represents depth in meters, and the x axis represents 16S rRNA gene copies per L. The area between the dash lines corresponds to the Oxygen Deficient Zone.

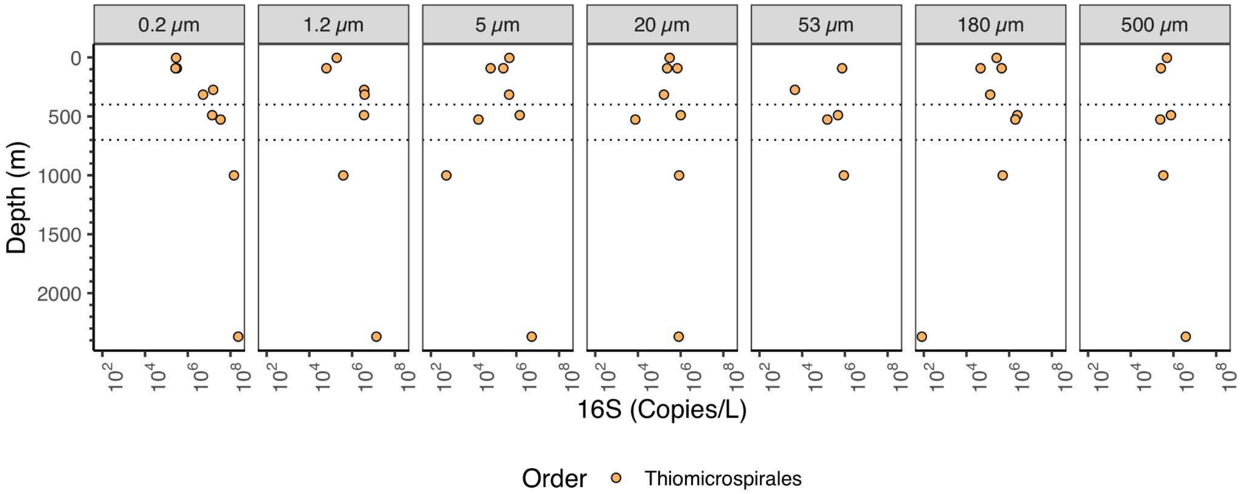

Supplementary Figure 8. Abundance of 16S gene copy numbers of Thioglobaceae aggregated to genus level. Each panel represents different particle sizes (in μm). The Y axis represents depth in meters, and the x axis represents 16S rRNA gene copies per L. The area between the dash lines corresponds to the Oxygen Deficient Zone.

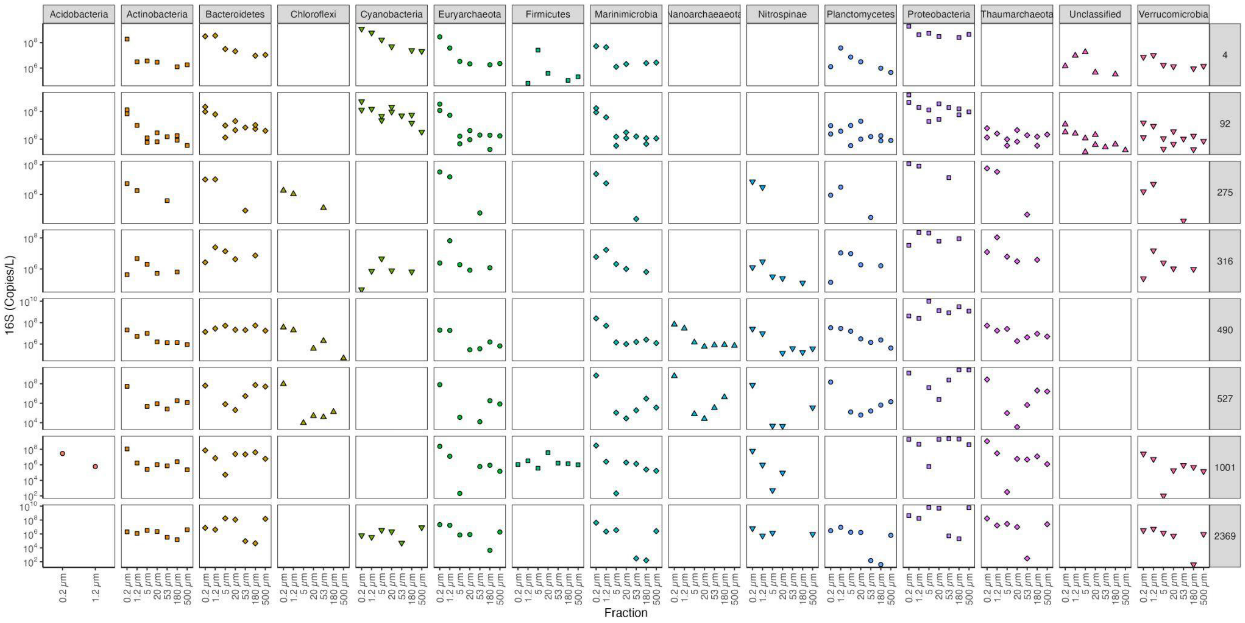

Supplementary Figure 9. Depth profile of 16S rRNA gene copy abundance of the top 10 most abundant Phyla for each depth. Data are normalized by volume. Each panel represents a particular phyla at a particular depth. The Y axis represents depth in meters, and the x axis represents 16S rRNA gene copies per L.

Supplementary Table 1. (Separate Document). Indicates the abundance, in 16s rRNA gene copies per liter of seawater, of the ten most abundant phylum level taxa, associated with each size class at each sampled depth. Depth, indicates the sampling

depth. Cast indicates the cast number. Fraction indicates the size fraction. Phylum indicates the Phylum level group. Copier per liter is the sum of the abundance of the copy number density of all ASVs in the given phylum, in each size class, at each sampling depth.
